# The LDH‐H3K18La‐Nur77 Axis Potentiates Immune Escape in Small Cell Lung Cancer

**DOI:** 10.1002/advs.202413608

**Published:** 2025-06-24

**Authors:** Xiaoling Shang, Bo Cheng, Chenyue Zhang, Chenglong Zhao, Ruiqing Wang, Xun Zhang, Dizhi Jiang, Xinyu Zhang, Xinyue Ma, Hongyuan Mao, Zerun Li, Chenhan Huang, Tianzi Wang, Kaiyue Guo, Liwen Wang, Ning Tang, Haiyong Wang

**Affiliations:** ^1^ Department of Internal Medicine‐Oncology Shandong Cancer Hospital and Institute Shandong First Medical University and Shandong Academy of Medical Sciences Jinan 250017 China; ^2^ Department of Radiation Oncology Qilu Hospital of Shandong University 107 Wenhuaxi Road Jinan 250012 China; ^3^ Department of Integrated Therapy Fudan University Shanghai Cancer Center Shanghai Medical College Shanghai 200032 China; ^4^ Department of Pathology The First Affiliated Hospital of Shandong First Medical University and Shandong Provincial Qianfoshan Hospital Jinan Shandong 250000 China; ^5^ Department of Medical Oncology University of Groningen, University Medical Center Groningen Hanzeplein 1 Groningen 9713 GZ The Netherlands

**Keywords:** H3K18La, lactate, naïve CD8^+^T cells, Nur77, SCLC

## Abstract

Small cell lung cancer (SCLC) remains a therapeutic challenge due to its aggressive nature and limited response to immunotherapy. This study identifies lactate‐induced histone lactylation as a novel epigenetic mechanism in SCLC, contributing to immune escape and poor therapeutic outcomes. By identifying the LDH‐H3K18La‐Nur77 axis, new insights into the metabolic regulation of immune responses in SCLC are offered. Multi‐omics analysis, including metabolomics and TCR sequencing, is used to compare serum samples and immune cell profiles from SCLC patients. Data from Shanzhong cohort (n = 222), along with a validation cohort from the IMpower133 study (n = 264), demonstrates that LDH levels are associated with poorer outcomes following immunotherapy. ChIP‐qPCR and luciferase reporter assays reveal that lactate‐induced histone lactylation at H3K18La induces transcriptional activation of Nur77 in naïve CD8^+^ T cells, leading to tonic TCR signaling, which impairs antigen recognition and prevents effective anti‐tumor activity. In preclinical SCLC models, lactate inhibition reduces Nur77 expression, restores T cell function, and enhances the efficacy of PD‐1 blockade, leading to a decreased tumor burden and improved survival. This study uncovers a novel mechanism of immune escape in SCLC through lactate‐driven histone lactylation and provides the first evidence that targeting lactate metabolism can enhance immunotherapy effectiveness.

## Graphical abstract

Lactate from SCLC tumors induces H3K18 lactylation in naïve CD8^+^ T cells, upregulating Nurr77 and enhancing tonic TCR signaling. This leads to T cell hyporesponsiveness to stimulation, impairing antitumor immunity and reducing immunotherapy efficacy. Lactate inhibition reverses this suppression restoring T cell function and improving immunotherapy response.

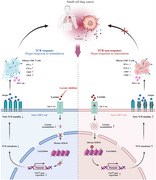

## 1 Introduction

Extensive‐stage small cell lung cancer (ES‐SCLC) presents a significant clinical challenge due to its aggressive progression and limited treatment modalities.^[^ ^1^, ^2^ ^]^ Despite the historical reliance on chemotherapy and radiotherapy, survival outcomes remain poor. In recent years, immunotherapy has emerged as a potential breakthrough, with immune checkpoint inhibitors (ICIs) targeting the PD‐1/PD‐L1 and CTLA‐4 pathways showing promise in improving survival for some ES‐SCLC patients. However, the overall response rates and durability of these responses remain suboptimal, emphasizing the necessity for novel therapeutic strategies.^[^ ^3^, ^4^, ^5^ ^]^

An increasing body of research has highlighted the complex interactions between tumor metabolism and immune regulation.^[^ ^6^, ^7^, ^8^, ^9^, ^10^ ^]^ Among the metabolic pathways altered in cancer, lactate metabolism, driven by the Warburg effect, is particularly prominent.^[^ ^8^, ^9^, ^10^ ^]^ This metabolic shift leads to excessive lactate production even in aerobic conditions, facilitating tumor proliferation while fostering an immunosuppressive tumor microenvironment (TME). Elevated lactate levels suppress immune function by altering the pH of the TME, thus impairing the activity of cytotoxic T lymphocytes (CTLs) and dendritic cells.^[^ ^11^, ^12^, ^13^ ^]^ Although targeting lactate metabolism holds considerable promise for improving immunotherapy outcomes, it remains an area of active investigation and has yet to be fully translated into clinical practice.

In addition to its metabolic functions, lactate acts as a substrate for histone lactylation, a novel post‐translational modification with profound implications for gene regulation. Histone lactylation has been implicated in immune evasion by modulating gene expression in immune cells within the TME.^[^ ^14^ ^]^ Studies indicate that this modification can promote the transcription of genes that suppress immune activity, aiding tumors in evading immune surveillance.^[^ ^15^, ^16^, ^17^ ^]^ For instance, histone lactylation can promote the expression of genes involved in the suppression of immune responses, thereby aiding tumor cells in evading immune surveillance.^[^ ^17^ ^]^ Furthermore, histone lactylation has been shown to influence immune cell differentiation and function, including the activation and cytotoxicity of CD8^+^ T cells, suggesting a potential mechanism for immunotherapy resistance.

The precise mechanisms by which histone lactylation influences immune responses are still being elucidated. However, it is becoming clear that this modification can regulate the expression of immune‐related genes, thereby impacting the immune landscape within the tumor. Thus, histone lactylation emerges as a promising epigenetic target for reprogramming immune cell function. Its modulation may help dismantle tumor‐induced immunosuppression and improve responsiveness to immunotherapeutic interventions.

A critical component of the immune response against tumors involves the activation and regulation of T cells. In this regard, the tonic signaling of T cell receptors (TCRs) on naïve CD8^+^T cells plays a significant role.^[^ ^18^, ^19^ ^]^ Tonic signaling refers to the baseline level of signaling in the absence of antigen engagement, which is essential for maintaining T cell survival and readiness.^[^ ^20^, ^21^ ^]^ Recent studies suggest that tonic TCR signals can influence the differentiation and functional capacity of effector T cells, potentially impacting their anti‐tumor activities.^[^ ^22^ ^]^ Modulating these tonic signals might enhance the effector functions of T cells, such as cytotoxicity and cytokine production, thereby improving the overall immune response against tumors. Further complicating this landscape are the downstream signaling molecules involved in T cell activation, including transcription factors such as Nur77.^[^ ^23^, ^24^, ^25^ ^]^ Nur77 is a critical regulator of T cell activation and apoptosis, and its expression is often used as a marker of TCR engagement. The modulation of Nur77 and related pathways could potentially enhance the specificity and effectiveness of T cell‐mediated immune responses against tumors.

In this study, we found that in SCLC, elevated lactate levels induced upregulation of Nur77 expression in naïve CD8^+^T cells through H3K18La lactylation modification, resulting in relatively low responsiveness to agonist TCR ligand stimulation, thereby weakening the overall anti‐tumor immune response. While immunotherapy has revolutionized the treatment landscape for SCLC, there is a clear need for innovative strategies to improve patient outcomes. The intersection of tumor metabolism, specifically lactate metabolism and histone lactylation, with immune regulation presents a promising area of research. Additionally, advancing our understanding of tonic TCR signaling on naïve CD8^+^T cells, along with the role of Nur77 in T cell responses, could offer new avenues for enhancing the efficacy of immunotherapies, which may lead to the development of more effective and targeted treatments for patients with ES‐SCLC and other malignancies.

## 2 Results

### 2.1 Elevated Lactate Levels Associated with Poor Immunotherapy Outcomes in ES‐SCLC Patients

**Figure 1:**
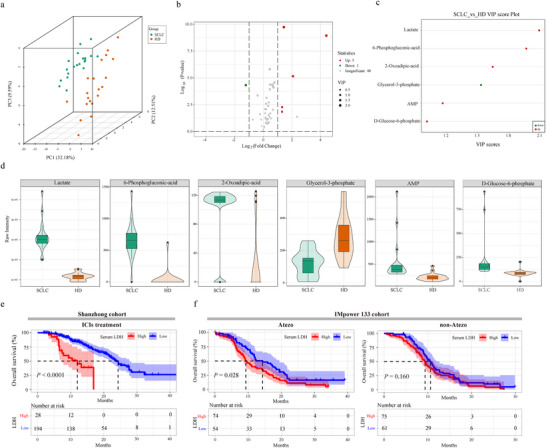
Metabolomic and prognostic significance of lactate and LDH in SCLC patients. a) A principal component analysis (PCA) of serum‐targeted metabolomics data comparing small cell lung cancer (SCLC) patients and healthy donors (HD). b) Volcano plot of the detected metabolites in serum metabolomics (SCLC patients versus HD). Significantly differential metabolites are colored in red (upregulated) and green (downregulated); the others are colored in gray. c) The VIP score of identified differential metabolites. d) Violin plots comparing metabolite levels between SCLC patients and HD. e) Kaplan‐Meier survival curves showing the overall survival (OS) of extensive‐stage small cell lung cancer (ES‐SCLC) patients from Shandong Cancer Hospital and Institute (Shanzhong) cohort treated with immune checkpoint inhibitors (ICIs) stratified by serum LDH levels. f) Kaplan‐Meier survival curves showing the OS of ES‐SCLC patients from IMpower133 cohort treated with atezolizumab (Atezo) or with non‐Atezo stratified by serum LDH levels.

The experimental design is summarized in Figure S1 (Supporting Information). Initial metabolomic profiling of serum samples from 20 treatment‐naïve SCLC patients and 20 healthy donors (HD) revealed significant metabolic reprogramming in SCLC, with lactate showing the most pronounced elevation (**Figure** **1**a–d). When stratifying these 20 SCLC patients by immunotherapy treatment response, we found significantly higher serum lactate levels in non‐responders compared to responders (Figure S2a–d, Supporting Information). This consistent pattern of lactate accumulation – observed both in SCLC patients versus HD and in non‐responders versus responders within the same patient cohort – suggests that lactate plays a pivotal role in mediating immune evasion and treatment resistance in SCLC.

Lactate dehydrogenase (LDH) is a commonly used marker in clinical peripheral blood tests and a key enzyme in the glycolytic pathway, responsible for converting pyruvate to lactate, suggesting that LDH levels are closely associated with lactate production. To further explore this association, we analyzed serum lactate and LDH levels in peripheral blood samples from 90 ES‐SCLC patients who received first‐line anti‐PD‐1/PD‐L1 immunotherapy combined with etoposide‐platinum (EP) chemotherapy at Shandong Cancer Hospital. Serum lactate and LDH levels for all 90 patients are summarized in Table S1 (Supporting Information). A strong positive correlation was observed between lactate and LDH (Spearman R = 0.835, *p* < 0.001; Figure S2e, Supporting Information), suggesting that LDH may reflect systemic lactate accumulation in these patients.

To further investigate the association between lactate metabolism and immunotherapy efficacy, we performed a retrospective cohort study of 222 ES‐SCLC patients treated with ICIs plus chemotherapy at Shandong Cancer Hospital. Baseline clinical characteristics (including age, sex, and serum LDH levels) are detailed in Table S2 (Supporting Information). Then, all patients were stratified into high and low LDH groups. Notably, patients with high LDH levels demonstrated significantly inferior response rates to ICIs compared to those with low LDH (*p* < 0.0001), suggesting a potential link between LDH‐mediated metabolic dysregulation and immunotherapy response in SCLC patients (Figure 1e). Furthermore, both univariate (HR = 4.285; 95% CI: 2.349 – 7.817; *p* < 0.001) and multivariate (HR = 4.037; 95% CI: 2.212 – 7.368; *p* < 0.001) cox regression analyses indicated that high LDH levels were a negative prognostic factor for overall survival (OS) in ES‐SCLC patients treated with ICIs (Table S3, Supporting Information). These findings further suggest that LDH‐mediated lactate metabolic dysregulation may contribute to immune evasion in ES‐SCLC.

To validate these findings, we utilized data from the IMpower133 study, which included information on 403 ES‐SCLC patients. After excluding patients with missing data, 264 patients were ultimately included in this study. The patients' characteristics are shown in Table S4 (Supporting Information). In patients treated with atezolizumab, similar to the results from the Shanzhong cohort, we observed that patients with high LDH levels had poorer outcomes compared to those with low LDH levels (*p =* 0.028). Interestingly, this stratification did not affect the outcomes of patients who received chemotherapy alone (*p =* 0.160), further suggesting that LDH specifically influences the efficacy of immunotherapy (Figure 1f). Univariate and multivariate analyses revealed that number of metastasis sites (HR = 1.909; 95% CI: 1.181–3.084; *p =* 0.008), and LDH levels (HR = 1.534; 95% CI: 1.025–2.298; *p =* 0.038) were independent and significant prognostic factors for OS in ES‐SCLC patients receiving atezolizumab treatment. The univariate and multivariate analyses of prognostic factors on OS are shown in Table S5 (Supporting Information). Additionally, based on LDH levels, all patients were classified into high‐ and low‐LDH groups. The survival curves revealed that atezolizumab treatment did not improve prognosis compared to chemotherapy in patients with high LDH levels (*p =* 0.17) (Figure S3a, Supporting Information). Interestingly, for ES‐SCLC patients with low LDH levels, those treated with atezolizumab had significantly better survival than those who received chemotherapy (*p =* 0.033) (Figure S3b, Supporting Information). More importantly, univariate and multivariate analyses indicated that atezolizumab treatment was a significant and independent prognostic factor for those with low LDH levels (HR = 0.597; 95% CI: 0.388–0.918; *p =* 0.019) (Table S6, Supporting Information).

Previous studies have reported that PD‐L1 expression may serve as a potential predictive biomarker for immunotherapy efficacy in SCLC.^[^ ^26^, ^27^ ^]^ To further evaluate potential biomarkers for immunotherapy response in SCLC, we analyzed PD‐L1 tumor proportion score (TPS) in 72 available tumor specimens from the Shanzhong cohort (n = 222). Immunohistochemical staining revealed PD‐L1 positivity (TPS ≥ 1%) in 11 patients (15.28%) and negativity in 61 patients (84.72%) (Figure S3c,d, Supporting Information). Survival analysis demonstrated no significant difference in immunotherapy outcomes between PD‐L1 TPS‐positive and ‐negative subgroups (*p =* 0.38) (Figure S3e, Supporting Information). This finding was validated in the IMpower133 cohort, where PD‐L1 expression status similarly failed to predict clinical benefit from atezolizumab treatment (*p =* 0.40) (Figure S3f, Supporting Information). In contrast to PD‐L1 expression, serum LDH levels provide a clinically accessible, quantitative, and reproducible biomarker that effectively stratifies SCLC patients likely to benefit from immunotherapy.

### 2.2 Lactate Modulates TCR Signaling in Naïve CD8^+^ T Cells via Nur77 Expression Enhancement

**Figure 2:**
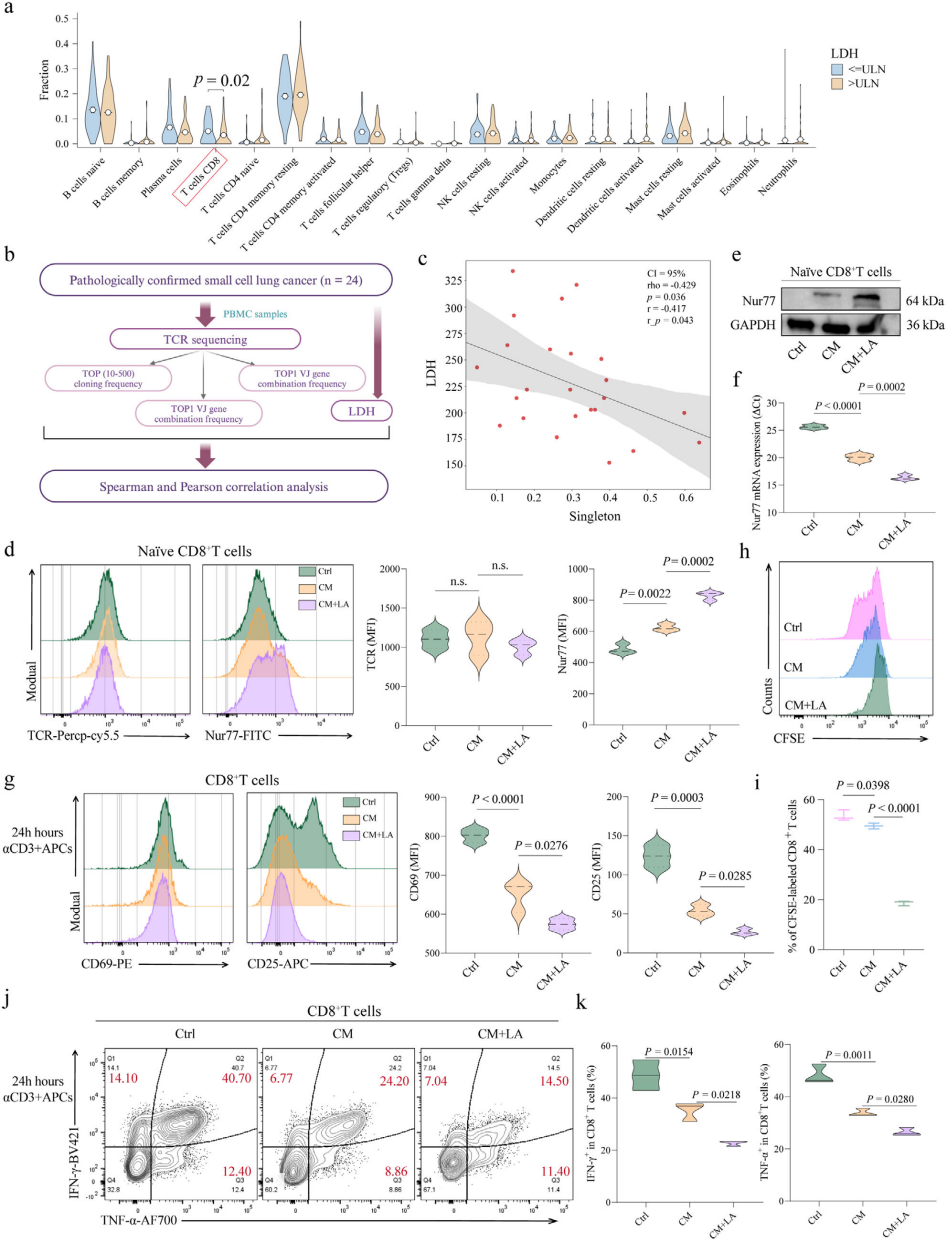
Extensive tonic TCR signaling and naïve CD8^+^ T cell responsiveness negatively correlate with elevated LDH levels. a) CIBERSORT analysis of RNA‐seq data from the IMpower133 cohort. b) Schematic workflow of TCR sequencing (TCRseq) performed on peripheral blood mononuclear cells (PBMCs) from 24 SCLC patients. c) The correlation between LDH expression and the TCR singleton index. d) Flow cytometry analysis of Nur77 and TCR expression in naïve CD8^+^ T cells after exposure to conditioned medium (CM) from H446 with or without lactate. e,f) Western blotting and RT‐qPCR analysis of the Nur77 levels in naïve CD8^+^ T cells after exposure to CM from H446 with or without lactate. g) Histograms show expression of the indicated activation markers of cells stimulated for 24 h with anti‐CD3 (0.25 µg mL^−1^) and APCs in CD8^+^ T cells after exposure to CM from H446 with or without lactate. Cells were gated on viable CD8^+^ T cells. h,i) Representative Contour plots and violin diagrams show the percentage of proliferated CFSE‐labeled T cells after exposure to CM from H446 with or without lactate. j,k) Contour plots and violin diagrams depict the secretion of IFN‐γ and TNF‐α by CD8^+^ T cells after exposure to CM from H446 with or without lactate. Data, mean ± S.E.M. of three independent experiments. n.s., not significant.

Initially, we utilized CIBERSORT to analyze bulk RNA‐seq data from IMpower133 cohort who received atezolizumab, assessing the infiltration levels of various immune cell types. Among the different immune cell populations analyzed, CD8^+^ T cell infiltration exhibited the most significant difference between high and low LDH expressing patients. Specifically, patients with high LDH levels demonstrated a marked reduction in CD8^+^ T cell infiltration (*p =* 0.02) (**Figure** **2**a). This finding suggests that elevated LDH may contribute to an immunosuppressive environment, potentially explaining the diminished efficacy of immunotherapy in these patients.

To further investigate the mechanisms behind the poor immunotherapy outcomes in SCLC patients with high LDH levels, we conducted TCR sequencing on peripheral blood mononuclear cells (PBMCs) from 24 SCLC patients. The experimental workflow is detailed in Figure 2b. The analysis revealed a negative correlation between LDH expression and the TCR singleton index, which indicates the proportion of TCR clones that appear only once (rho = −0.429, *p =* 0.036) (Figure 2c). A higher singleton index is typically associated with a greater proportion of naïve T cells, which are crucial for recognizing new antigens. Therefore, we hypothesized that increased LDH levels in SCLC patients might induce naïve CD8^+^ T cells exhibited relative hypo‐responsiveness to stimulation with agonist TCR ligands, thereby weakening the overall anti‐tumor immune response.

Several molecules have been implicated in the regulation of tonic TCR signaling, among which CD5 and Nur77 are two of the most extensively characterized and functionally relevant markers.^[^ ^28^, ^29^, ^30^ ^]^ To elucidate the key regulatory factors governing tonic TCR signaling within the SCLC microenvironment, we performed targeted knockdown of CD5 and Nur77 in naïve CD8⁺ T cells. We then evaluated TCR signaling activity and assessed the capacity of these cells to secrete TNF‐α and IFN‐γ following antigen stimulation. Our data revealed that knockdown of Nur77 significantly enhanced the functional activation of naïve CD8⁺ T cells, as evidenced by increased cytokine production (Figure S4a,b, Supporting Information), whereas CD5 knockdown did not elicit a comparable effect (Figure S4c,d, Supporting Information). Furthermore, flow cytometric analysis of naïve CD8⁺ T cells isolated from healthy donors and treated with control medium, conditioned medium (CM) from H446, CM supplemented with lactate, or CM with a lactate inhibitor (LAi), showed no appreciable change in CD5 expression (Figure S5a,b, Supporting Information). Collectively, these findings highlight Nur77 as a pivotal regulator of tonic TCR signaling and underscore its potential role in modulating antitumor immune responses in the SCLC microenvironment.

To validate the hypothesis, CD8^+^ T cells from healthy volunteers were isolated and exposed to different conditions: control medium, CM from SCLC cell lines (H446, H69, H82) with or without lactate. Flow cytometry analysis demonstrated that Nur77 expression in naïve CD8^+^ T cells was significantly upregulated in both the CM and lactate groups compared to the control (Figure 2d; Figure S6a,b, Supporting Information). Increased Nur77 expression in naïve T cells reflects the frequency and intensity of relatively recently experienced tonic TCR signaling. Further supporting these findings, western blot and PCR analyses confirmed the significant increase in Nur77 expression under the same conditions (Figure 2e,f).

Subsequent experiments assessed the differentiation of naïve CD8^+^ T cells into effector T cells. Upon stimulation with CD3 and other antigens, the expression of activation markers CD69 and CD25 was found to be suppressed in the CM and lactate groups compared to the control group (Figure 2g; Figure S6c,d, Supporting Information). Additionally, the functional analysis revealed a significant decrease in CD8^+^ T cell proliferation and the secretion of key cytokines, IFN‐γ and TNF‐α, in these groups (Figure 2h–k, Figure S6e, Supporting Information). These results suggest that lactate‐induced tonic TCR signaling significantly suppresses the proliferation and differentiation of naïve CD8^+^ T cells.

### 2.3 Lactate Inhibition Partially Rescues the Responsiveness of Nur77‐Driven Naïve CD8⁺ T Cells in SCLC

**Figure 3:**
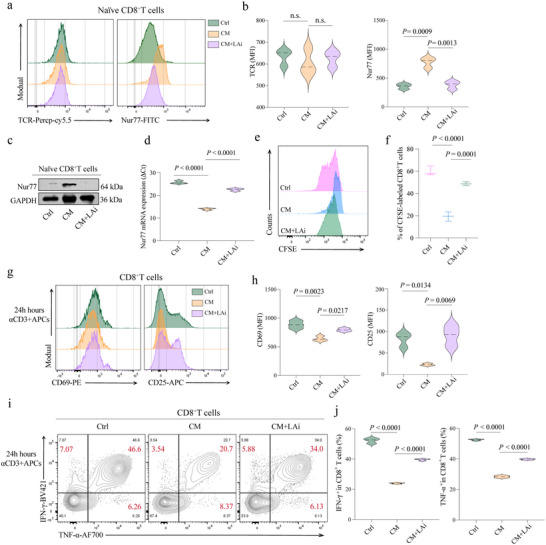
Effects of lactate inhibition on Nur77 expression and functional reprogramming of naïve CD8^+^ T cells in SCLC. a) Flow cytometry analysis of Nur77 and TCR expression in naïve CD8^+^ T cells after exposure to CM from H446 with or without lactate inhibitor (LAi). b) Violin diagrams display the mean fluorescence intensity (MFI) for Nur77 and TCR. c,d) Western blotting and RT‐qPCR analysis of the Nur77 protein levels in naïve CD8^+^ T cells after exposure to CM from H446 with or without LAi. e,f) Representative histograms and scatter graphs show the percentage of proliferated CFSE‐labeled T cells after exposure to CM from H446 with or without LAi. g) Histograms show expression of the indicated activation markers of cells stimulated for 24 h with anti‐CD3 (0.25 µg mL^−1^) and APCs in CD8^+^ T cells after exposure to CM from H446 with or without LAi. Cells were gated on viable CD8^+^ T cells. h) Violin diagrams display the MFI for CD69 and CD25. i,j) Contour plots and violin diagrams depict the secretion of IFN‐γ and TNF‐α by CD8^+^ T cells after exposure to CM from H446 with or without LAi. Data, mean ± SEM of three independent experiments. n.s., not significant.

To explore the potential therapeutic impact of inhibiting lactate on reversing the responsiveness of Nur77‐mediated naïve CD8^+^ T cells in SCLC, the naïve CD8^+^ T cells were exposed to different treatment conditions, including a LAi. Flow cytometric analysis was utilized to quantify the expression of Nur77 and the TCR on naïve CD8^+^ T cells incubated under different conditions. Interestingly, our results demonstrated that while the LAi had no significant effect on TCR expression, it led to a pronounced downregulation of Nur77 on naïve CD8^+^ T cells (**Figure** **3**a,b; Figure S7a,b, Supporting Information). Next, we conducted additional analyses using western blot and PCR, both of which confirmed the reduction in Nur77 expression on naïve CD8^+^ T cells following treatment with the LAi (Figure 3c,d). The decrease in Nur77 suggests a reduction in tonic signaling, which is often associated with a more quiescent state in T cells. This reduction in tonic signaling could potentially enhance the cells' responsiveness to new antigens, providing a more adaptable immune response.

Given the observed suppression of tonic TCR signaling by lactate, we next examined whether lactate inhibition could restore the differentiation of naïve CD8^+^ T cells into functional effector cells and enhance their antitumor activity. We investigated the functional consequences of Nur77 downregulation by assessing the proliferative capacity of CD8^+^ T cells using a CFSE assay. The results demonstrated a significant improvement in CD8^+^ T cell proliferation in the LAi group (Figure 3e,f). Additionally, we also assessed the expression of activation markers CD69 and CD25. The increased expression of these markers, accompanied by an increased secretion of key cytokines, TNF‐α and IFN‐γ, in the LAi‐treated group, indicated a heightened state of T cell activation (Figure 3g–j; Figure S7c–e, Supporting Information). Taken together, these sequential experiments demonstrate that lactate inhibition in the SCLC leads to decreased Nur77 expression in naïve CD8^+^T cells, reduces TCR tonic signaling, and subsequently enhances T cell activation and effector function.

### 2.4 Lactate Inhibition Potentiates the Therapeutic Effect of PD‐1 Blockade

**Figure 4:**
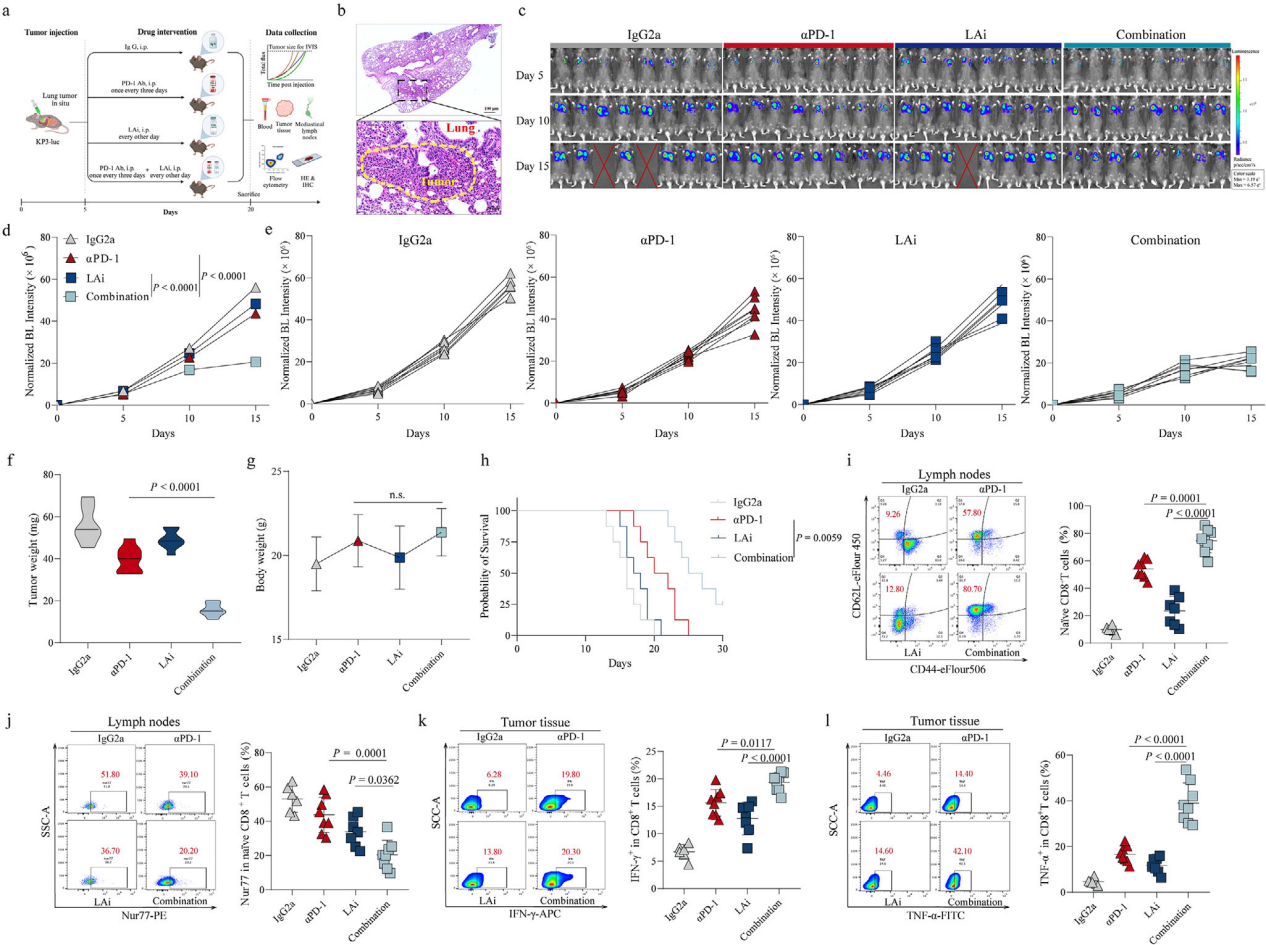
Therapeutic effects of combining lactate inhibition with αPD‐1 therapy in an orthotopic SCLC mouse model. a) Schematic representation of the four experimental groups: control, αPD‐1, LAi, and combination of αPD‐1 and LAi. b) H&E staining of the orthotopic lung tumor model in mice. c) In vivo bioluminescent images of four groups at the indicated time points. d,e) Bioluminescence intensity was measured at 5 days post‐injection of luciferase‐expressing KP3 cells into mouse lungs, comparing four different treatment groups. f) Comparison of tumor weight across treatment groups. g) Comparison of body weight in mice across four groups. h) Kaplan‐Meier survival analysis of the mice in different treatment groups. i) The mediastinal lymph nodes were analyzed by flow cytometry for naïve CD8^+^ T cells (CD44^low^CD62L^high^). Cells were gated on viable CD8^+^ T cells. j) The mediastinal lymph nodes were analyzed by flow cytometry for Nur77 expression on naïve CD8^+^ T cells. k,l) Orthotopic tumors were analyzed by flow cytometry for IFN‐γ and TNF‐α expression on CD8^+^ T cells. Scatter graphs represent the means ± S.E.M. for each group. c–k) n = 8 for each group. One‐way ANOVA statistical tests were adopted for more than two groups. Log‐rank tests were used in h. n.s., not significant.

To build upon the previous findings that suggested lactate's involvement in the remodeling of the immune microenvironment, we sought to evaluate the therapeutic potential of combining lactate inhibition with PD‐1 antibody therapy in the context of SCLC. By inhibiting lactate production, we aimed to investigate whether this could enhance the efficacy of immune checkpoint blockade with PD‐1 antibodies. To test this hypothesis, we first established an orthotopic lung tumor model in mice. This model closely mimics the tumor's natural environment and is therefore an appropriate system for evaluating therapeutic interventions. The mice were subsequently divided into four experimental groups, as depicted in **Figure** **4**a, to receive different treatments: αPD‐1, LAi alone, a combination of αPD‐1 and LAi, or a control treatment. The H&E staining confirmed the successful establishment of the orthotopic lung tumor model, providing a reliable basis for further experimentation (Figure 4b). Quantitative assessment of tumor volume was conducted using fluorescence imaging of live animals, a method that allows for precise and non‐invasive monitoring of tumor progression over time. The results revealed that the combination of αPD‐1 with the LAi resulted in a significant reduction in tumor volume, indicating a synergistic effect between these two therapeutic strategies (Figure 4c–e). Furthermore, tumor weight was also significantly lower in the combination treatment group compared to the groups receiving either treatment alone (Figure 4f). Consistently, IHC staining showed that combination therapy with LAi and αPD‐1 synergistically suppressed Ki67^+^ tumor cells (Figure S8a,b, Supporting Information). LAi significantly decreased lactate content in tumors compared to the corresponding controls (Figure S8c, Supporting Information). Importantly, no significant differences in body weight were observed among the treatment groups throughout the study period, suggesting that the LAi was well‐tolerated and did not induce significant toxicity in the animals (Figure 4g). Importantly, mice receiving the combination therapy exhibited a substantial extension in survival time, underscoring the enhanced therapeutic efficacy of this approach (Figure 4h).

To further elucidate the impact of the combination therapy on the TME, we examined the mediastinal lymph nodes from terminal‐stage mice (Figure S8d, Supporting Information). Specifically, we assessed the number of naïve CD8^+^ T cells and their expression of Nur77. The flow gate strategies of lymph nodes and tumor tissue are shown in Figure S8e,f (Supporting Information). The results demonstrated that the combination therapy significantly increased the number of naïve CD8^+^ T cells (CD44^low^CD62L^high^) in the mediastinal lymph nodes, while simultaneously downregulating Nur77 expression (Figure 4i,j). Similarly, the results of peripheral blood flow in mice also confirmed this finding (Figure S8g,h, Supporting Information). Additionally, we analyzed the functionality of CD8^+^ T cells within the tumor tissue to assess the direct impact of the combination therapy on anti‐tumor immune responses. The data showed that CD8^+^ T cells in the combination therapy group had a markedly enhanced capacity to secrete key cytokines, including IFN‐γ and TNF‐α, both of which are essential for effective anti‐tumor immunity (Figure 4k,l). The IHC staining also demonstrated CD8^+^ T cells showed increased infiltration in the combination therapy group compared to other treatment group (Figure S8i, Supporting Information). The increased secretion of these cytokines indicates that the combination of lactate inhibition and PD‐1 blockade not only reduces tumor burden but also revitalizes the immune system's ability to fight cancer.

### 2.5 H3K18La Induces Transcriptional Activation of Nur77 in Naïve CD8^+^ T Cells

**Figure 5:**
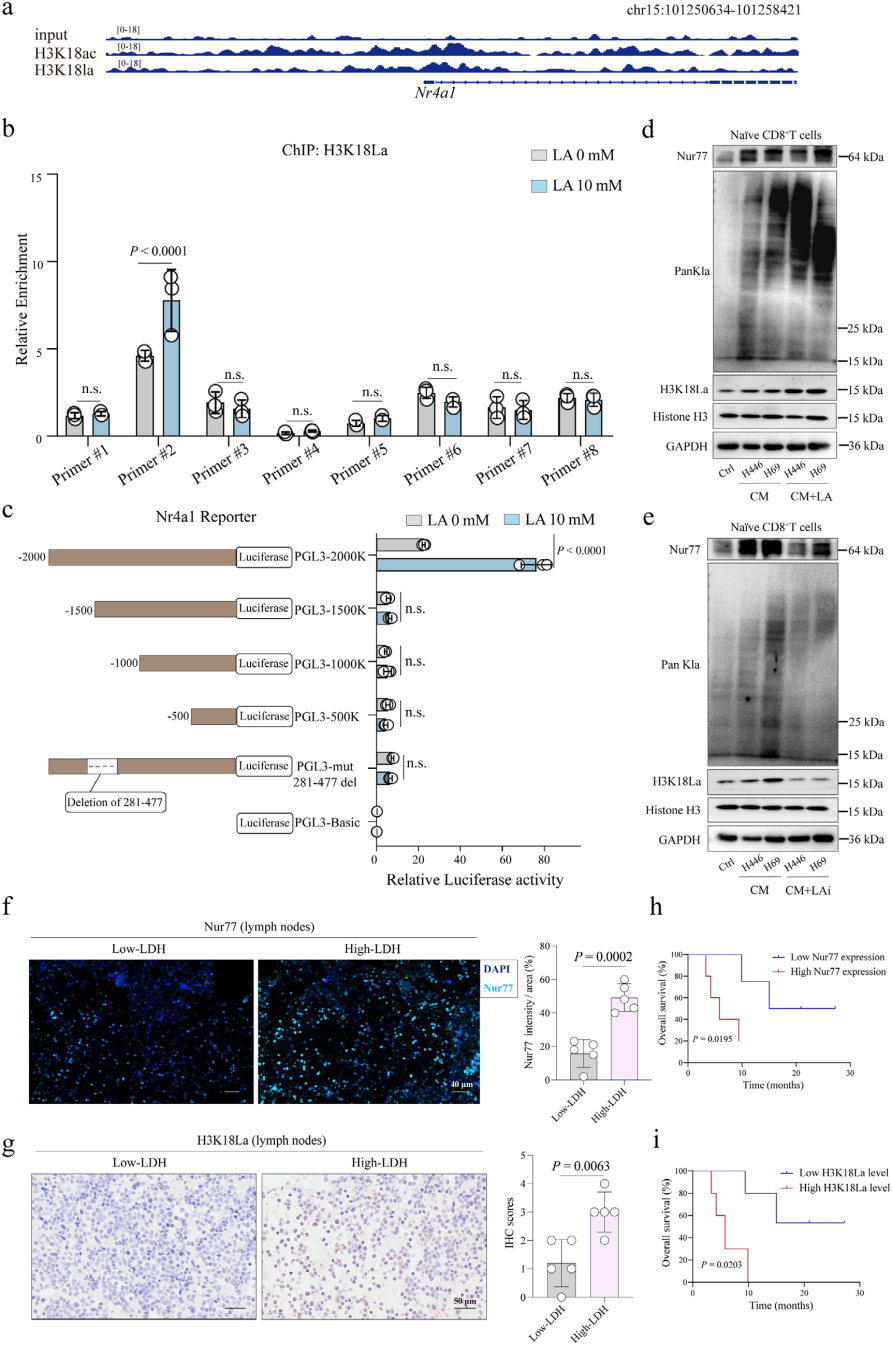
Role of histone lactylation in regulating Nur77 expression and its clinical implications in SCLC. a) IGV tracks for Nur77 from ChIP‐seq were shown. b) ChIP‐qPCR was conducted on naïve CD8^+^ T cells treated with LA (0 mm, 10 mm) for 24 h. c) Deletion analysis identified H3K18La‐enrichment region in the Nur77 promoter (left panel), along with the relative luciferase activity measured (right panel). d) Western blotting analysis of PanKla, H3K18La and Nur77 levels in naïve CD8^+^ T cells under H446, H69 CM or LA conditions. e) Western blotting analysis of PanKla, H3K18La and Nur77 levels in naïve CD8^+^ T cells under H446, H69 CM or LAi conditions. f) Representative images of Immunofluorescence staining for Nur77 expression in lymph nodes of ES‐SCLC patients (n = 10) stratified by LDH. Scale bars: 40 µm. g) Representative images of IHC staining for H3K18La expression in lymph nodes of ES‐SCLC patients (n = 10) stratified by LDH. Scale bars: 50 µm. h,i) The OS curves of ES‐SCLC patients (n = 10) from Shandong Cancer Hospital with low and high Nur77 g) or H3K18La h) levels were generated using Kaplan‐Meier method and the log‐rank test.

H3K18La, as a novel epigenetic modification, has been found to directly stimulate gene transcription by altering chromatin structure. In the mouse orthotopic lung tumor model, we analyzed the lactylation levels in various cell types within the SCLC TME (Figure S9a, Supporting Information). The results showed that pan‐lactylation levels were significantly higher in tumor cells and CD8^+^ T cells compared to CD4^+^ T cells and tumor‐associated macrophages (TAMs). Notably, CD8^+^ T cells exhibited a significantly more pronounced lactylation at the H3K18 site. This finding suggests that lactate‐driven histone lactylation, particularly at the H3K18 site in CD8^+^ T cells, may play a critical role in regulating their function and potentially contribute to immune evasion in the SCLC microenvironment. To further investigate the potential role of lactate in regulating Nur77 expression, we reviewed existing studies that highlighted the significance of histone lactylation in gene transcription regulation. Additionally, we reanalyzed previously published ChIP‐seq data to determine whether H3K18La is enriched at the Nur77 gene promoter.^[^ ^14^ ^]^ Analysis with ChIP‐seeker showed obvious enrichment of H3K18La peaks at the Nur77 promoter region, as shown in **Figure** **5**a. Next, we designed eight potential binding regions for H3K18 at the Nur77 promoter and synthesized corresponding primers. ChIP‐qPCR results showed that, upon treatment with 10 mm lactate, H3K18La was significantly enriched in the 281–477 bp region of the Nur77 promoter, while no such enrichment was observed in the other regions (Figure 5b). Moreover, we further conducted a series of luciferase reporter assays using truncated versions of the Nur77 promoter, spanning regions −2000, −1500, −1000, and −500 kb, and the specific 281–477 bp deletion region. Our results revealed that H3K18La primarily enriched at the 281–477 bp region of the Nur77 promoter (Figure 5c). This confirms that the 281–477 bp region is the key binding region for H3K18La, supporting the conclusion that H3K18La induces transcriptional activation of Nur77 in naïve CD8^+^ T cells. Next, naïve CD8^+^ T cells were cultured in CM derived from SCLC cell lines (H446, H69, and H82) treated with lactate. The results showed a marked increase in H3K18La levels and Nur77 expression in the naïve CD8^+^ T cells (Figure 5d; Figure S9b, Supporting Information). However, when the CM was derived from cells treated with LAi, this effect was significantly reversed (Figure 5e; Figure S9c, Supporting Information), reinforcing the role of lactate in modulating these epigenetic and transcriptional changes.

To extend these findings to a clinical context, we analyzed lymph node tissue samples from 10 patients with SCLC. The samples were stratified into high‐LDH and low‐LDH groups based on LDH levels, a marker of lactate production. Immunofluorescence analysis revealed that patients in the high‐LDH group exhibited significantly elevated Nur77 expression (Figure 5f). Moreover, IHC staining indicated that H3K18La levels were also increased in the high‐LDH group (Figure 5g). Survival analysis further substantiated the clinical relevance of these findings, showing that high levels of Nur77 and H3K18La were associated with shorter OS in SCLC patients treated with immunotherapy (Figure 5h,i). These results suggest that lactate‐driven histone lactylation at H3K18La promotes Nur77 transcription, leading to poor prognosis in patients receiving immunotherapy.

### 2.6 Targeting LDH‐H3K18La‐Nur77 axis Enhances Anti‐PD‐1 Efficacy

**Figure 6:**
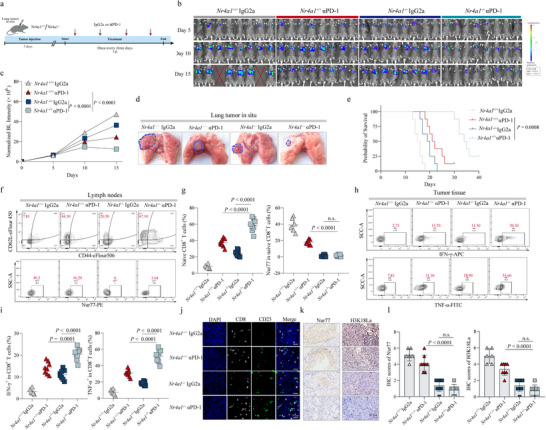
NR4A1 ablation remodels the immunosuppressive microenvironment to enhance anti‐PD‐1 efficacy in SCLC. a) Schematic representation of the experimental design: *Nr4a1* ^−/−^ and *Nr4a1* ^+/+^ mice treated with αPD‐1 or IgG2a isotype control. b) In vivo bioluminescent images of mice from different treatment groups. c) Quantitative analysis of bioluminescence intensity after injection of luciferase‐expressing KP3 cells into the lungs. d) Representative images of lung orthotopic tumors in four groups. e) Kaplan‐Meier survival analysis of the mice in different treatment groups. f) Flow cytometry analysis of naïve CD8^+^ T cells and Nur77^+^naïve CD8^+^ T cells in mediastinal lymph nodes of mice. g) Scatter graphs show the frequencies of naïve CD8^+^ T cells and Nur77^+^naïve CD8^+^ T cells in mediastinal lymph nodes of mice from different treatment groups. h) Flow cytometry analysis of IFN‐γ and TNF‐α expression on CD8^+^ T cells in tumor tissues of mice. i) Scatter graphs show the frequencies of IFN‐γ, TNF‐α‐secreting cells in orthotopic tumors. j) Representative images of Immunofluorescence staining for CD8^+^CD25^+^ cells in tumor tissues of mice from different treatment groups. CD8: white, CD25: green, Scale bars, 50 µm. k) Representative images of IHC staining of Nur77 and H3K18La in tumor tissues of mice from different treatment groups. Scale bars, 50 µm. l) Quantitative analysis of Nur77 and H3K18La expression. b–j) n = 8 for each group. One‐way ANOVA statistical tests were adopted for more than two groups. Log‐rank tests were used in e. n.s., not significant.

To mechanistically validate the role of Nur77 in lactate‐mediated immune suppression within the SCLC microenvironment, we generated a CD8⁺ T cell‐specific Nur77 conditional knockout mouse model (hereafter referred to as *Nr4a1* ^−/−^). Wild‐type littermates (*Nr4a1* ^+/+^) served as controls. An orthotopic lung tumor model was established by implanting SCLC cells (KP3) directly into the lungs of both groups. Mice were then treated with either anti‐PD‐1 antibody (αPD‐1) or an IgG2a isotype control, as illustrated in **Figure** **6**a. Remarkably, *Nr4a1* ^−/−^ mice treated with αPD‐1 exhibited a pronounced reduction in tumor burden and extended OS, accompanied by significantly lower Ki67 expression in lung tumors compared to control mice (Figure 6b–e; Figure S10a, Supporting Information). These results indicate an enhanced anti‐tumor response resulting from CD8⁺ T cell‐specific ablation of Nur77. Complementary biochemical assays revealed reduced lactate levels in *Nr4a1* ^−/−^ mice following αPD‐1 administration (Figure S10b, Supporting Information), consistent with disruption of the LDH‐histone lactylation‐Nur77 axis. Flow cytometric analysis of mediastinal lymph nodes and peripheral blood further showed an increased proportion of naïve CD8⁺ T cells, key mediators of durable anti‐tumor immunity, in *Nr4a1* ^−/−^ mice treated with αPD‐1. As anticipated, these CD8⁺ T cells exhibited markedly diminished Nur77 expression, confirming successful gene knockout (Figure 6f,g; Figure S10c,d, Supporting Information). Concomitantly, the TME of *Nr4a1* ^−/−^ mice treated with αPD‐1 showed elevated levels of IFN‐γ and TNF‐α (Figure 6h,i), indicative of a heightened pro‐inflammatory immune response. Immunofluorescence staining of lung tumor tissues demonstrated increased infiltration of total CD8⁺ T cells and activated CD25⁺CD8⁺ T cells in the *Nr4a1* ^−/−^ mice following αPD‐1 group (Figure 6j; Figure S10e, Supporting Information). Moreover, immunohistochemical analysis revealed substantial decreases in both Nur77 and H3K18La levels within tumor‐infiltrating CD8⁺ T cells (Figure 6k,l), underscoring the CD8⁺ T cell‐intrinsic nature of the LDH‐H3K18La‐Nur77 pathway. Collectively, these findings identify Nur77 as a critical downstream effector of lactate‐driven immune suppression in SCLC and demonstrate that targeting the LDH–H3K18La–Nur77 axis enhances the therapeutic efficacy of PD‐1 blockade by reprogramming CD8⁺ T cell function in the TME.

### 2.7 Correlation Between Nur77 Expression and Anti‐PD‐1 Therapy Efficacy in SCLC Patients

**Figure 7:**
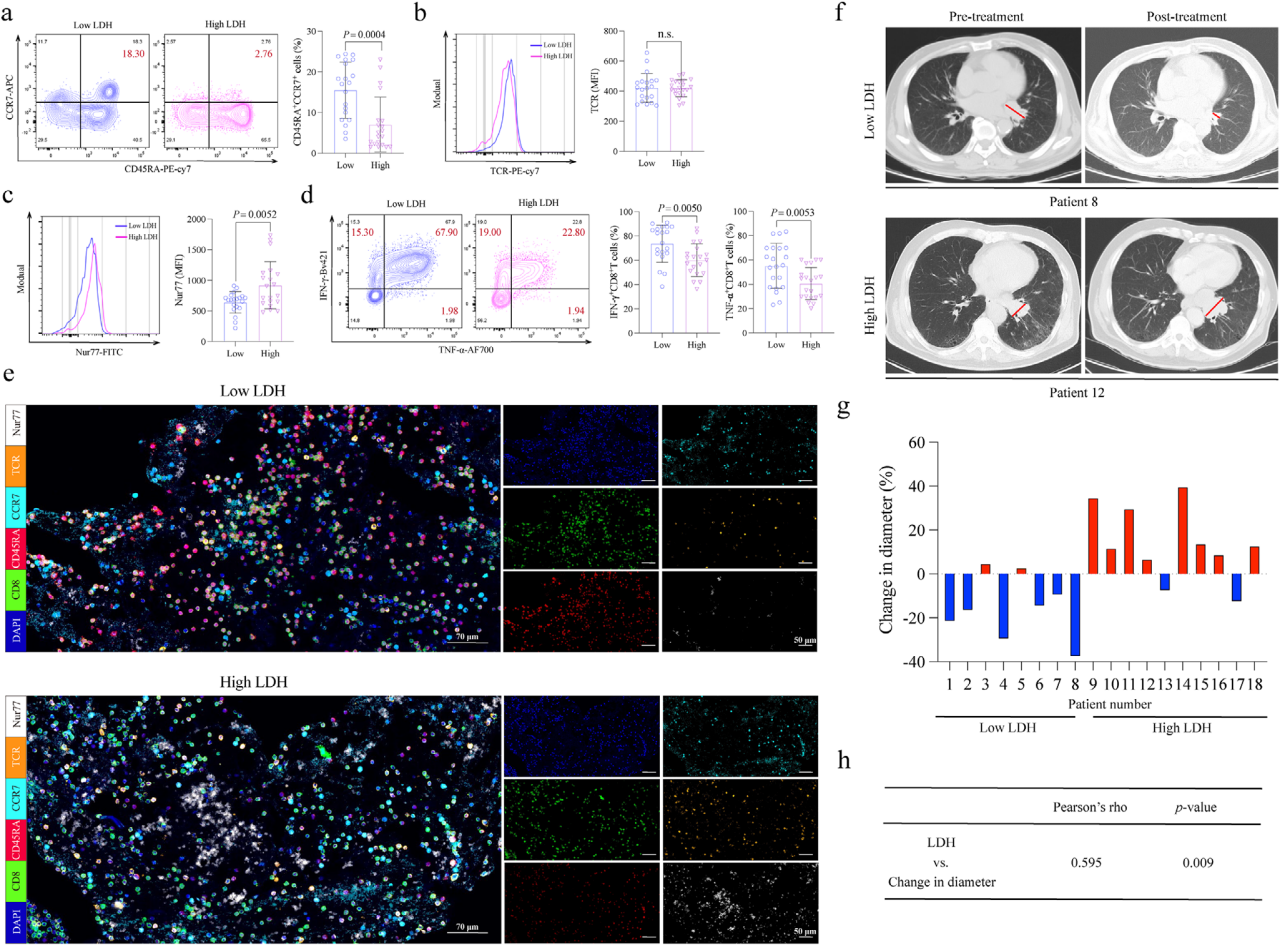
The LDH‐Nur77 axis modulates immune checkpoint therapy efficacy in ES‐SCLC. a) Flow cytometry analysis shows the number of naïve CD8^+^ T cells (CD45RA^+^ CCR7^+^) in the peripheral blood (PB) of ES‐SCLC patients stratified by LDH levels. b,c) Histograms and graph bars show expression of TCR and Nur77 on naïve CD8^+^ T cells in the PB of ES‐SCLC patients. d) Contour plots and graph bars depict the secretion of IFN‐γ and TNF‐α by CD8^+^ T cells in the PB of ES‐SCLC patients. e) Representative images of multiplex immunohistochemistry (mIF) staining of CD8, CD45RA, CCR7, TCR and Nur77 expression in lymph nodes between ES‐SCLC patients with low‐ and high‐LDH level. Scale bars, 70 µm, left panel; 50 µm, right panel. f) Two ES‐SCLC patients treated with anti‐PD‐1 therapy representing a responder (Pt.8) and non‐responder (Pt.12) case were analyzed. Tumor diameter based on the CT imaging was annotated by the radiologist with a red line. g) The difference in tumor diameter for 18 patients from Qilu Hospital of Shandong University, where those with increased tumor diameter are colored by red. h) Quantitative correlation between the change in tumor diameter and LDH levels. The correlation coefficient and the p values were computed based on Pearson method. n.s., not significant.

To further investigate the role of the LDH‐H3K18La‐Nur77 axis in modulating the efficacy of ICIs therapy in SCLC, we conducted a comprehensive analysis involving 40 patients treated with ICIs. The study cohort was stratified into two groups based on serum LDH levels: 20 patients with high LDH levels and 20 with low LDH levels. Initially, we assessed the quantity of naïve CD8^+^ T cells in the peripheral blood of these patients, along with the expression levels of Nur77 and TCR. The results revealed a significant reduction in the number of naïve CD8^+^ T cells (CD45RA^+^ CCR7^+^) in patients with high LDH levels (**Figure** **7**a). This observation prompted us to further examine the signaling through TCR and the expression of Nur77 on these naïve CD8^+^ T cells. Our analysis showed that Nur77 expression was markedly upregulated in the naïve CD8^+^ T cells of high‐LDH patients, while TCR expression levels remained relatively unchanged (Figure 7b,c). Given these findings, we next evaluated the functional capacity of CD8^+^ T cells in terms of cytokine secretion. Specifically, we measured the production of IFN‐γ and TNF‐α in CD8^+^ T cells from both high‐LDH and low‐LDH patients (Figure 7d). The results indicated that the CD8^+^ T cells from high‐LDH patients exhibited significantly diminished secretion of these key cytokines, suggesting an impaired anti‐tumor immune response. To further validate these observations, we conducted multiplex immunofluorescence (mIF) analysis on lymph node tissues obtained from both high‐LDH and low‐LDH SCLC patients. This analysis focused on quantifying the infiltration of naïve CD8^+^T cells and assessing the expression of TCR and Nur77. Consistent with our earlier findings, LDH levels were inversely correlated with the number of naïve CD8^+^T cells in lymph node tissues and positively correlated with Nur77 expression (Figure 7e). These correlations suggest a potential mechanistic link between elevated LDH levels, enhanced Nur77 expression and reduced T‐cell efficacy.

Among the 18 ES‐SCLC patients receiving ICIs treatment from Qilu Hospital of Shandong University, we observed that 8 patients responded positively to the treatment, while the remaining 10 exhibited poor responses. A detailed case analysis highlighted two representative patients: Patient 8, who had low LDH levels and experienced significant tumor shrinkage following therapy, and Patient 12, who had high LDH levels and whose tumor continued to progress despite treatment (Figure 7f). Across all patients, a strong positive correlation was observed between tumor diameter and LDH expression levels blockade (rho = 0.595, *p =* 0.009) (Figure 7g,h). Overall, these findings suggest that the LDH‐H3K18La‐Nur77 axis could be a potential predictor of response to PD‐1 antibody therapy in SCLC. Elevated LDH levels, which are associated with increased Nur77 expression and impaired T‐cell function, may contribute to resistance to immunotherapy. Therefore, targeting this axis could improve the therapeutic outcomes for patients with SCLC.

## 3 Discussion

**Figure 8:**
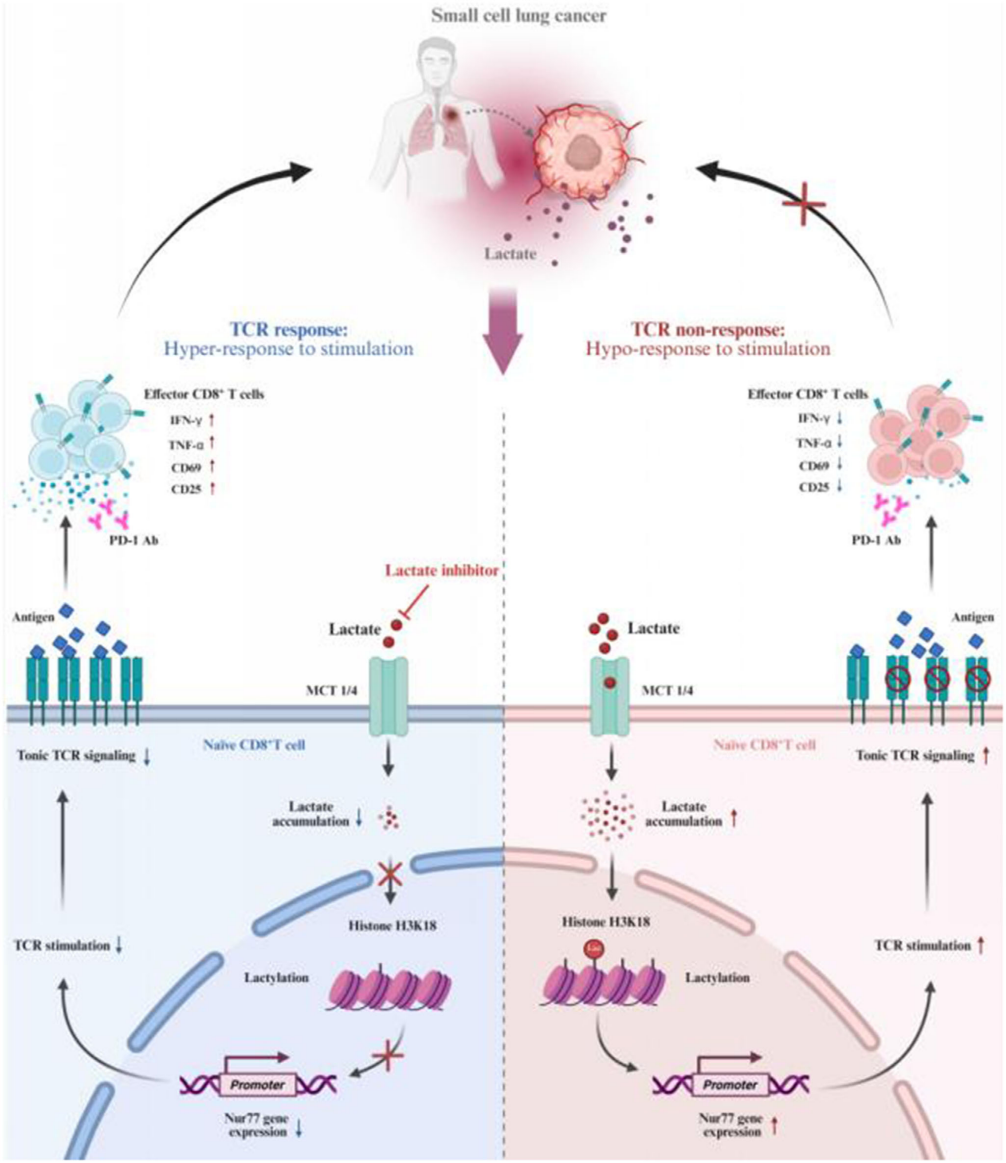
Model of the proposed mechanism of LDH‐H3K18La‐Nur77 axis. SCLC tumor‐derived lactate initiates a signaling cascade in naïve CD8^+^ T cells resulting in Histone H3K18 lactylation dependent up‐regulation of Nur77 expression, inducing increased levels of tonic TCR signaling. These naïve CD8^+^ T cells were hyporesponsive to agonist TCR stimulation, inhibiting antitumor T cell activity, and thus limits the efficacy of immunotherapy. Inhibition of lactate with lactate inhibitor will overcome lactate‐mediated immunosuppression and sensitize tumors to immunotherapy.

This study uncovers several groundbreaking findings in the context of SCLC and its metabolic interactions with the immune system, particularly emphasizing the critical role of lactate. For the first time, we demonstrate that elevated lactate levels in SCLC significantly influence immune responses by driving histone lactylation and regulating TCR signaling through Nur77 expression. This novel mechanism of lactate‐induced histone lactylation, specifically at the H3K18La site, not only impairs CD8^+^ T cell activation but also facilitates immune escape in SCLC (**Figure** **8**). Furthermore, we reveal that targeting lactate metabolism enhances the efficacy of PD‐1 blockade, offering a promising therapeutic strategy. Notably, this study takes a from clinical observation to clinical solution approach, addressing the challenge of immunotherapy resistance in SCLC by uncovering its metabolic and immune mechanisms and proposing a promising combination of lactate inhibition with PD‐1 blockade. This highlights the translational relevance and potential real‐world impact of our findings. These discoveries fill a crucial gap in understanding how metabolic shifts within the TME directly contribute to immune escape in SCLC and represent a significant leap forward in the potential treatment options for this aggressive cancer.

Recent work emphasizes lactate's role in modulating the TME. Elevated lactate levels can inhibit CD8^+^ T cell cytotoxicity by altering metabolic pathways, contributing to immune evasion in various cancers.^[^ ^31^, ^32^, ^33^ ^]^ This study demonstrates that elevated lactate levels, driven by increased LDH activity, play a central role in shaping the immunosuppressive TME in SCLC. By linking LDH expression to the poor outcomes of immunotherapy, we highlight lactate as a critical factor in promoting immune escape. The Shanzhong cohort and the validation cohort from IMpower133 cohort both show that patients with high LDH levels exhibit significantly reduced survival when treated with ICIs. These results are consistent across cohorts, underscoring the robustness of the findings and the relevance of LDH as a biomarker for predicting immunotherapy efficacy. Importantly, this study goes beyond observational data by demonstrating a direct mechanistic link between lactate metabolism and immune regulation, specifically through the modulation of TCR signaling in CD8^+^ T cells.^[^ ^34^, ^35^ ^]^

It has been established that lactate accumulation in the TME promotes tumor growth, angiogenesis, and immune escape by influencing the behavior of immune cells. This suppression is mediated by lactate‐driven acidosis and lactylation, which hinder immune cell function and recruitment.^[^ ^36^, ^37^ ^]^ Lactate influences the immune response by upregulating Nur77 expression in naïve CD8^+^ T cells, leading to tonic TCR. This form of signaling hinders T cell activation and proliferation, weakening the overall anti‐tumor response signaling.^[^ ^38^, ^39^, ^40^ ^]^ The reduction in cytokine secretion, such as IFN‐γ and TNF‐α, further exemplifies the immune escape facilitated by lactate in the SCLC microenvironment. By inhibiting lactate production, our study demonstrates that TCR signaling is normalized, allowing for more robust CD8^+^ T cell activation and improving the immune response. These findings reveal an intricate metabolic‐immune interaction, where lactate modulates immune function at a cellular and molecular level, contributing directly to tumor immune escape.

In addition to clarifying the role of lactate in immune escape, this study provides the first evidence that lactate inhibition, combined with PD‐1 blockade, can significantly improve anti‐tumor immunity in SCLC. In preclinical models, we show that combination therapy reduces tumor burden and extends survival, highlighting the synergistic potential of targeting metabolic pathways alongside immune checkpoint blockade. The increased infiltration of activated CD8^+^ T cells in the TME and the enhanced secretion of key cytokines support the therapeutic promise of this approach. These results suggest that lactate inhibition may provide a new avenue for overcoming immune escape and improving the efficacy of immunotherapy in SCLC patients.

Further mechanistic exploration revealed that lactate‐induced histone lactylation at H3K18La is directly involved in Nur77 transcriptional regulation. This epigenetic modification serves as a novel mechanism by which lactate promotes immune escape,^[^ ^41^, ^42^, ^43^ ^]^ driving sustained Nur77 expression and impairing the anti‐tumor immune response. Importantly, we show that this lactate‐driven histone lactylation can be reversed with lactate inhibitors, which, in turn, reduce Nur77 expression and restore T‐cell functionality. This epigenetic regulation of immune cells represents a significant breakthrough in understanding how metabolic changes in the TME contribute to immune escape and resistance to therapy.

While our study primarily focuses on the immunosuppressive effects of H3K18La in CD8^+^ T cells, we also detected this modification in tumor cells, albeit at lower levels (Figure S9a, Supporting Information). This observation suggests a dual regulatory role for histone lactylation within the TME, operating through both immune cell modulation and tumor cell‐autonomous mechanisms. In tumor cells, H3K18La may contribute to immune evasion and therapy resistance by enhancing glycolytic metabolism (Warburg effect),^[^ ^44^ ^]^ thereby sustaining a lactate‐rich milieu that further promotes histone lactylation across cellular compartments. This self‐reinforcing cycle could maintain immune checkpoint molecule expression through lactylation‐mediated transcriptional activation while simultaneously altering the secretion of immunomodulatory cytokines. Additionally, H3K18La may coordinate chemokine expression to recruit immunosuppressive myeloid cells, establishing parallel immune escape pathways, as similar to H3K18 lactylation‐induced immunosuppression in NSCLC.^[^ ^17^ ^]^ These findings highlight the synergistic interplay between tumor cell‐intrinsic lactylation and immune cell dysfunction, providing new insights into the aggressive and treatment‐resistant nature of SCLC. Future studies should aim to identify specific gene targets of H3K18La in tumor cells and elucidate their crosstalk with immune cell lactylation networks, which could inform the development of combined metabolic and epigenetic therapeutic strategies.

Inhibitors of lactate production or transport, such as LDH inhibitors or MCT1/4 inhibitors, have been found to potentiate immunotherapy in NSCLC and hepatocellular carcinoma.^[^ ^45^, ^46^ ^]^ Additionally, metabolic reprogramming strategies, including dietary modifications or pharmacological agents that promote lactate clearance, may help reshape the TME to favor immune activation. Future research should focus on identifying patient subgroups that would benefit most from targeting lactate metabolism, as well as understanding potential resistance mechanisms. Biomarker‐driven clinical trials incorporating lactate‐related signatures may help guide treatment selection. Furthermore, investigating the broader impact of lactate metabolism on other immune and stromal cells within the TME will be essential for developing comprehensive therapeutic strategies. Therefore, targeting lactate metabolism presents a promising avenue for improving immunotherapy outcomes in SCLC patients. By integrating metabolic targeting with immunotherapy, we may overcome the intrinsic resistance of SCLC and improve patient outcomes.

In this study, we observed a significant correlation between elevated LDH activity, increased histone H3K18la, and reduced expression of the nuclear receptor Nur77 in naïve CD8⁺ T cells of SCLC. Although this LDH‐H3K18la‐Nur77 axis has not been fully elucidated, it may represent a novel metabolic‐epigenetic‐immune regulatory pathway contributing to immune dysfunction in the TME. Previous landmark studies have shown that lactate, a byproduct of glycolysis catalyzed by LDH, can serve as a donor substrate for histone lactylation.^[^ ^47^, ^48^ ^]^ This post‐translational modification is potentially mediated by histone acetyltransferases such as p300/CBP,^[^ ^14^ ^]^ linking metabolic flux to chromatin remodeling and gene expression. However, the mechanistic link between H3K18la and the transcriptional repression of Nur77 remains to be defined. One possible explanation is that H3K18la may alter chromatin accessibility at the Nur77 promoter region, thereby impairing the recruitment of essential transcriptional activators. Alternatively, lactylation may influence the binding of specific transcription factors or co‐regulators that modulate Nur77 expression. Candidates such as HIF‐1α or members of the NR4A family, which are known to respond to metabolic and inflammatory signals, warrant further investigation.

Although this study has made significant strides in understanding the role of lactate metabolism in immune escape, there are limitations that warrant further investigation. The sample sizes, despite sufficient for this analysis, could benefit from larger multi‐center studies to validate the findings in more diverse patient populations. Additionally, while the preclinical models provide strong evidence for the combination of lactate inhibition and PD‐1 blockade, clinical trials are needed to assess the safety, optimal dosing, and timing of such therapies in human patients. Furthermore, the long‐term effects of lactate inhibition, particularly in relation to immune function and potential off‐target effects, should be closely monitored in future studies.

In summary, our study provides compelling evidence that targeting lactate metabolism significantly enhances the anti‐tumor efficacy of PD‐1 blockade in SCLC. This effect is achieved by inhibiting the level of H3K18La and subsequently downregulating the expression of Nur77, thereby alleviating the naïve CD8^+^ T cell TCR tonic signal and enhancing its responsiveness to antigen, which collectively enhances the activation and function of CTLs. These findings represent a significant advancement in our understanding of SCLC biology and pave the way for future therapeutic innovations targeting metabolic‐immune interactions.

## 4 Experimental Section

### Patient Samples

A total of 264 ES‐SCLC patients with known clinical information from IMpower133 study (IMpower133 cohort) and 222 ES‐SCLC patients from Shandong Cancer Hospital and Institute (Shanzhong cohort) were included in this study. The IMpower133 study is a randomized, double‐blind, phase I/III study, demonstrated that adding atezolizumab to carboplatin plus etoposide for first‐line treatment of ES‐SCLC resulted in significant improvement in OS versus placebo plus carboplatin and etoposide.^[^ ^49^ ^]^ All patients in this study provided written informed consent and this study was approved by the Ethics Committee of Shandong Cancer Hospital and Institute (Grant No. SDTHEC2024006145). It also conforms to the provisions of the Declaration of Helsinki. The recruited patients from both IMpower133 and Shanzhong cohorts were subject to further analyses.

### Cell Culture

Human SCLC cell lines, H446, H69, and H82, were purchased from the China Center for Type Culture Collection and maintained in RPMI 1640 (Gibco, USA) supplemented with 10–20% fetal bovine serum (FBS, Gibco). The Murine SCLC cell line KP3^[^ ^50^ ^]^ was purchased from ChengYin Biological Company (Shanghai, China) and cultured in DMEM (Gibco) with 10% FBS (Gibco). All cells were cultured at 37 °C humidified conditions with 5% CO2 and routinely tested for mycoplasma.

### Animal Study

All procedures involving animals were conducted in accordance with institutional and national guidelines for animal care and use. The health status of the animals was monitored daily, and efforts were made to minimize suffering. Prior to experimental procedures, mice were acclimated to the housing conditions for at least one week. Experimental protocols were approved by the Institutional Animal Care and Use Committee (IACUC). All mice were housed in a specific pathogen‐free (SPF) facility at a constant temperature of 22±2 °C, with a 12‐hour light/dark cycle. The mice were provided with standard chow and water ad libitum.

To generate CD8⁺ T cell‐specific Nur77 knockout mice (Nr4a1⁻/⁻), Nr4a1^fl/fl^ mice (C57BL/6 background) were crossed with CD8‐Cre transgenic mice (C57BL/6 background). The conditional knockout mice (Nr4a1^fl/fl^; CD8‐Cre) and their littermate wild‐type controls (Nr4a1^fl/fl^) were genotyped by PCR and housed under SPF conditions. All mice were used at 6–8 weeks of age. The conditional knockout strain was generated with the assistance of GemPharmatech Co., Ltd. (Nanjing, China). All animal experiments were conducted in compliance with the Institutional Animal Care and Use Committees of Shandong Cancer Hospital and Institute (Grant No. SDTHEC2022003100).

For orthotopic SCLC model establishment, the murine KP3 cell line was used. KP3 cells were cultured under standard conditions, harvested, and resuspended in sterile PBS at 5 × 10^5^ cells per 40 µL. Mice were anesthetized using isoflurane, and a small incision was made in the left thoracic region to expose the lung. Using a 30G insulin syringe, 5 × 10^5^ KP3 cells were injected into the left lung lobe. The incision was sealed with surgical glue, and mice were monitored daily for postoperative recovery. Tumor progression was assessed by survival analysis, histology, and subsequent immunological and molecular assays.

### Metabolic Analysis

Baseline serum samples were collected from 20 patients with ES‐SCLC who received ICIs in combination with chemotherapy. Age‐ and sex‐matched healthy donors (HD, n = 20) were included as controls. All serum samples were processed by centrifugation and stored at −80 °C prior to analysis. Targeted metabolomic profiling was performed in collaboration with Maiwei Metabolomics (Shanghai, China). Metabolites were extracted using a methanol‐based protocol and analyzed using liquid chromatography‐mass spectrometry (LC‐MS). Data acquisition and processing were performed using standardized workflows, and metabolites were identified based on mass spectra and retention time matching against a validated reference database. Based on treatment outcomes evaluated by RECIST v1.1 criteria, SCLC patients were stratified into responders and non‐responders. Statistical analyses, including univariate and multivariate approaches, were conducted to identify differentially expressed metabolites between SCLC patients and healthy controls, as well as between responders and non‐responders.

### TCR Sequencing

For T cell receptor sequencing, PBMCs were collected from 24 SCLC patients before anti‐tumor treatment. The blood was processed promptly, and PBMCs were isolated using standard density gradient centrifugation. The cells were cryopreserved for later analysis. Total RNA was isolated from PBMCs by RNeasy Plus Mini Kit (Qiagen, USA) and was synthesized into the cDNA library by iRepertoire Short Read iR‐Profile Reagent System HTBI‐v. The TCR β‐chain was amplified and sequenced using the Illumina MiSeq platform. Sequencing data were analyzed to identify V, D, and J gene segments and assess TCR diversity and clonality. Public and private clonotypes were identified by examining CDR3 regions.

### Estimation of Immune Cell Type Fractions

The ES‐SCLC bulk RNAseq data from IMpower133 were downloaded from the European Genome‐Phenome Archive (https://ega‐archive.org/studies). To quantify the abundance of immune cells in ES‐SCLC specimens, we applied CIBERSORT to provide an estimation of the proportions of cell types in a mixed cell population using normalized data. The infiltrating immune cells inferred by CIBERSORT include B cells, plasma cells, T cells, NK cells, monocytes, macrophages, dendritic cells, mast cells, eosinophils, and neutrophils.^[^ ^51^ ^]^

### Western Blotting

Protein concentrations were measured using BCA Protein Assay Kit (Thermo Fisher). Cell lysates were prepared from naïve CD8^+^T cells and separated by SDS–PAGE, and separated proteins were then transferred onto PVDF membranes (Millipore). Membranes were blocked with 5% milk in TBST (TBS + 0.1% Tween‐20) for 2 h at room temperature and incubated with primary antibodies overnight at 4 °C. The used primary antibodies are shown in Table S7 (Supporting Information). Subsequently, Membranes were washed three times with TBST, incubated with horseradish peroxidaseconjugated goat anti‐rabbit secondary antibodies (1:10 000) for 1 h at room temperature, washed three times with TBST again, and protein bands were visualized by ECL (Thermo Fisher).

### RNA Extraction and Real‐Time Quantitative PCR

Total RNA was extracted from cells with TRIzol reagent (Thermo Fisher) and reverse transcribed into cDNA by using the HiScript III RT SuperMix for quantitative PCR (Vazyme; Cat No. R323‐01). The mRNA expression of Nur77 in naïve CD8^+^ T cells was determined using specific primers and analyzed using the comparative Ct method and normalized to GAPDH level. The primer sequences were described in Table S8 (Supporting Information).

### The Multiplex Immunofluorescent (mIF) Staining

ES‐SCLC lymph node samples were obtained by biopsies. 4 um thick formalin‐fixed paraffin‐embedded (FFPE) tissue sections from Shanzhong cohort were evaluated with mIF technology. The staining was performed using the Opal 7‐Color IHC Kit (Akoya Biosciences, USA) and imaged by a PerkinElmer Vectra 3.0 (Perkin Elmer, Hopkington, MA) multispectral microscope. Specificity for each staining has been validated. The antibodies used for multiplex mIF staining were listed in Table S9 (Supporting Information).

### Immunohistochemical (IHC) Staining and Quantification

Human lymph node tissues and mouse tumor tissues were first embedded in paraffin and then sectioned into 4 µm slices. The sections were deparaffinized and rehydrated in a descending ethanol series. Following antigen retrieval, the sections were incubated with 3% hydrogen peroxide for 20 min. Tissue slides were then incubated overnight at 4 °C with primary antibodies. The primary antibodies used for IHC staining were shown in Table S10 (Supporting Information). The two‐step IHC kit (zsbio; Cat No. PV‐9000) was used for protein expression analysis, according to the manufacturer's instructions. Finally, the slides were visualized with the 3, 3′‐diaminobenzidine solution (DAB) and counterstained with hematoxylin.

### Flow Cytometry

Tumors were minced and digested with collagenase IV/DNase I, filtered, and resuspended in single‐cell suspension for FACS staining. Fluorophore‐conjugated antibodies against the indicated surface markers were used to stain single‐cell solutions of tumors and mediastinal lymph node cells. Cells were incubated for 20 min on ice with Viability Dye 780 (biogems) and antibodies against CD45, CD44, CD62L, Nur77, CD8. For cytokine staining, cells were stimulated with a cell‐stimulation cocktail plus protein transporter inhibitors (eBioscience) for 4 h, stained with surface antibodies, fixed, permeabilized, and stained with antibodies against IFN‐γ and TNF‐α. All antibodies were purchased from BioLegend or eBioscience. Stained cells were analyzed using the BD FACSymphony A3 Cell Analyzer flow cytometer, and further data analysis was performed using FlowJo software. The antibodies and reactive dye were listed in Table S11 (Supporting Information).

### Isolation of CD8^+^T Cells

Peripheral blood was collected from healthy volunteers using EDTA‐coated tubes. To isolate CD8^+^T cells, PBMCs were first separated using Ficoll‐Paque PLUS density gradient centrifugation. The isolated PBMCs were washed twice with phosphate‐buffered saline (PBS) and resuspended in MACS buffer (PBS containing 0.5% bovine serum albumin and 2 mm EDTA). CD8 T cells were then isolated from the PBMCs suspension using magnetic bead‐based selection. Specifically, the cells were resuspended in MACS buffer and labeled with the CD8 T cell Biotin‐Antibody Cocktail and Anti‐Biotin MicroBeads. After incubation and washing, the cell suspension was applied to an LS column in a MACS Separator. The magnetic field retained nonCD8 T cells labeled with MicroBeads, while labeled CD8 T cells were washed away. The column was then removed from the magnetic field, and the CD8 T cells were eluted, collected, centrifuged, and resuspended in a culture medium. The purity and viability of the isolated CD8^+^T cells were assessed by flow cytometry, confirming that the cells were over 95% pure. The isolated cells were then used for downstream analyses, such as functional assays and molecular studies. The antibodies were listed in Table S11 (Supporting Information).

For the T cell proliferation assay, anti‐CD3‐preactivated T cells were labeled with CFSE (5 µM) (Invitrogen; Cat No. C34554) for 20 minutes at 37 °C. The labeled cells were then washed thoroughly and divided into different treatment groups, followed by culture in complete RPMI medium. T cell proliferation was assessed by flow cytometry after 72 h based on CFSE dilution.

### ChIP‐qPCR

ChIP–qPCR was performed using the ChIP Assay Kit (Active Motif) according to the manufacturer's protocol. Briefly, naïve CD8^+^ T cells were fixed with 1% formaldehyde on ice to cross‐link the proteins bound to the chromatin DNA. After washing, the chromatin DNA was sheared by enzymatic force to produce DNA fragments of ≈200–1000 bp. The same amounts of sheared DNA were used for immunoprecipitation using anti‐L‐Lactyl‐Histone H3 (Lys18). The immunoprecipitate was then incubated with protein G magnetic beads, and the antibody‐protein G magnetic beads complex was collected for subsequent reverse cross‐linking. The same amount of sheared DNA without antibody precipitation was processed for reverse cross‐linking and served as input control. DNA recovered from reverse cross‐linking was used for qPCR. The ChIP–qPCR primers for the Nur77 promoter were listed in Table S12 (Supporting Information).

### Dual‐Luciferase Reporter Assay

To functionally validate the regulatory role of the H3K18La‐enriched region in Nur77 promoter activity, we performed dual‐luciferase reporter assays. Based on our ChIP‐qPCR results identifying the 281–477 bp region as the primary H3K18La binding site, we generated a series of truncated Nur77 promoter constructs: −2000, −1500, −1000, and −500 bp relative to the transcription start site (TSS), along with a specific deletion mutant lacking the 281–477 bp region. All constructs were cloned into the pGL3‐Basic vector (Promega) upstream of the firefly luciferase gene. For transfection, HEK293T cells were seeded in 24‐well plates and co‐transfected with 400 ng of each promoter construct and 20 ng of pRL‐TK Renilla luciferase control vector (Promega) using Lipofectamine 3000 (Invitrogen). At 24 h post‐transfection, cells were treated with 10 mM sodium lactate or vehicle control for an additional 24 h. Luciferase activity was measured using the Dual‐Luciferase Reporter Assay System (Promega) according to the manufacturer's protocol. Firefly luciferase activity was normalized to Renilla luciferase for each sample. Three independent experiments were performed in triplicate.

### Lactate and LDH Measurement

Lactate levels in patient serum and mouse tumor tissues were measured using a Lactate Assay Kit (ab65331, Abcam). For serum samples, 100 µL of each serum was incubated with the assay reagents following the manufacturer's instructions. Absorbance was measured at 450 nm using a microplate reader. For tumor tissue, 50 mg of tissue was homogenized in 1 mL of assay buffer, followed by centrifugation to collect the supernatant. Serum LDH levels were determined using a standard clinical biochemical method, where 100 µL of serum was mixed with LDH reagent, and the absorbance at 340 nm was measured to calculate enzyme activity.

### Statistical Methods

All data were expressed as mean ± SD. Multiple‐group comparisons in vivo and ex vivo assays were analyzed by Dunnett's multiple‐comparisons test (one‐way ANOVA), Sidak's multiple‐comparisons test (two‐way ANOVA), and Tukey's multiple‐comparisons test (two‐way ANOVA). A minimum of three independent results from cell experiments were evaluated. The differences between the two groups were verified using an unpaired Student t‐test and considered statistically significant when *p* < 0.05. R packages and GraphPad Prism 9.0 were used for statistical analysis.

## Conflict of Interest

The authors declare no conflict of interest.

## Author Contributions

X.S. and B.C. contributed equally to this work. H.W. performed project administration, conceptualization, resources, data curation, and funding acquisition; X.S. and B.C. wrote the original draft, reviewed, and edited the manuscript, performed supervision, methodology, in vivo and in vitro experiments; C.Z. and C.Z. performed conceptualization, methodology, validation; R.W. and X.Z. performed methodology, validation, resources, supervision; D.J., X.Z., and X.M. collected patient medical record and follow‐up, animal model construction; H.M. and Z.L. performed patient imaging data inquiry, efficacy evaluation; C.H. and T.W. performed statistical analysis, experiments, and animal feeding; K.G., L.W., and N.T. performed methodology, sample collection, software, visualization.

## Supporting information



Supporting Information

## Data Availability

The data that support the findings of this study are available from the corresponding author upon reasonable request.

## References

[1] Z. Megyesfalvi , C. M. Gay , H. Popper , R. Pirker , G. Ostoros , S. Heeke , C. Lang , K. Hoetzenecker , A. Schwendenwein , K. Boettiger , P. A. Bunn , F. Renyi‐Vamos , K. Schelch , H. Prosch , L. A. Byers , F. R. Hirsch , B. Dome , CA Cancer J. Clin. 2023, 73, 620. 37329269 10.3322/caac.21785

[2] W. J. Petty , L. Paz‐Ares , JAMA Oncol. 2023, 9, 419. 36520421 10.1001/jamaoncol.2022.5631

[3] B. Y. Nabet , H. Hamidi , M. C. Lee , R. Banchereau , S. Morris , L. Adler , V. Gayevskiy , A. M. Elhossiny , M. K. Srivastava , N. S. Patil , K. A. Smith , R. Jesudason , C. Chan , P. S. Chang , M. Fernandez , S. Rost , L. M. McGinnis , H. Koeppen , C. M. Gay , J. D. Minna , J. V. Heymach , J. M. Chan , C. M. Rudin , L. A. Byers , S. V. Liu , M. Reck , D. S. Shames , Cancer Cell 2024, 42, 429. 38366589 10.1016/j.ccell.2024.01.010

[4] J. Zugazagoitia , H. Osma , J. Baena , A. C. Ucero , L. Paz‐Ares , Clin. Cancer Res. 2024, 30, 2872. 38630789 10.1158/1078-0432.CCR-23-1159

[5] W. T. Iams , J. Porter , L. Horn , Nat. Rev. Clin. Oncol. 2020, 17, 300. 32055013 10.1038/s41571-019-0316-z PMC7212527

[6] M. De Martino , J. C. Rathmell , L. Galluzzi , C. Vanpouille‐Box , Nat. Rev. Immunol. 2024, 24, 654. 38649722 10.1038/s41577-024-01026-4 PMC11365797

[7] D. Wilfahrt , G. M. Delgoffe , Nat. Immunol. 2024, 25, 206. 38238609 10.1038/s41590-023-01733-5

[8] Z. Zong , F. Xie , S. Wang , X. Wu , Z. Zhang , B. Yang , F. Zhou , Cell 2024, 187, 2375. 38653238 10.1016/j.cell.2024.04.002

[9] R. Ding , X. Yu , Z. Hu , Y. Dong , H. Huang , Y. Zhang , Q. Han , Z.‐Y. Ni , R. Zhao , Y. Ye , Q. Zou , Immunity 2024, 57, 528. 38417442 10.1016/j.immuni.2024.01.019

[10] K. Chaudagar , H. M. Hieromnimon , A. Kelley , B. Labadie , J. Shafran , S. Rameshbabu , C. Drovetsky , K. Bynoe , A. Solanki , E. Markiewicz , X. Fan , M. Loda , A. Patnaik , Clin. Cancer Res. 2023, 29, 4930. 37721526 10.1158/1078-0432.CCR-23-1441 PMC10841690

[11] J. Ma , L. Tang , Y. Tan , J. Xiao , K. Wei , X. Zhang , Y. Ma , S. Tong , J. Chen , N. Zhou , L. Yang , Z. Lei , Y. Li , J. Lv , J. Liu , H. Zhang , K. Tang , Y. Zhang , B. Huang , Nat. Immunol. 2024, 25, 552. 38263463 10.1038/s41590-023-01738-0 PMC10907288

[12] S. Verma , S. Budhu , I. Serganova , L. Dong , L. M. Mangarin , J. F. Khan , M. A. Bah , A. Assouvie , Y. Marouf , I. Schulze , R. Zappasodi , J. D. Wolchok , T. Merghoub , J. Clin. Invest. 2024, 134. 10.1172/JCI177606 PMC11364391 39225102

[13] M. P. Plebanek , Y. Xue , Y.‐V. Nguyen , N. C. DeVito , X. Wang , A. Holtzhausen , G. M. Beasley , B. Theivanthiran , B. A. Hanks , Sci. Immunol. 2024, 9, adi4191. 10.1126/sciimmunol.adi4191 PMC11926670 38728412

[14] D. Zhang , Z. Tang , H. Huang , G. Zhou , C. Cui , Y. Weng , W. Liu , S. Kim , S. Lee , M. Perez‐Neut , J. Ding , D. Czyz , R. Hu , Z. Ye , M. He , Y. G. Zheng , H. A. Shuman , L. Dai , B. Ren , R. G. Roeder , L. Becker , Y. Zhao , Nature 2019, 574, 575. 31645732 10.1038/s41586-019-1678-1 PMC6818755

[15] A. De Leo , A. Ugolini , X. Yu , F. Scirocchi , D. Scocozza , B. Peixoto , A. Pace , L. D'Angelo , J. K. C. Liu , A. B. Etame , A. Rughetti , M. Nuti , A. Santoro , M. A. Vogelbaum , J. R. Conejo‐Garcia , P. C. Rodriguez , F. Veglia , Immunity 2024, 57, 1105. 38703775 10.1016/j.immuni.2024.04.006 PMC11114377

[16] Z.‐W. Huang , X.‐N. Zhang , L. Zhang , L.‐L. Liu , J.‐W. Zhang , Y.‐X. Sun , J.‐Q. Xu , Q. Liu , Z.‐J. Long , Signal. Transduct. Target Ther. 2023, 8, 391. 37777506 10.1038/s41392-023-01605-2 PMC10542808

[17] C. Zhang , L. Zhou , M. Zhang , Y. Du , C. Li , H. Ren , L. Zheng , Cancer Res. 2024, 84, 3589. 39137401 10.1158/0008-5472.CAN-23-3513

[18] B. D. Hale , Y. Severin , F. Graebnitz , D. Stark , D. Guignard , J. Mena , Y. Festl , S. Lee , J. Hanimann , N. S. Zangger , M. Meier , D. Goslings , O. Lamprecht , B. M. Frey , A. Oxenius , B. Snijder , Science 2024, 384, adh8697. 10.1126/science.adh8967 38843327

[19] A. Straub , S. Grassmann , S. Jarosch , L. Richter , P. Hilgendorf , M. Hammel , K. I. Wagner , V. R. Buchholz , K. Schober , D. H. Busch , Immunity 2023, 56, 1269e6. 37164014 10.1016/j.immuni.2023.04.010

[20] A. H. Courtney , W. L. Lo , A. Weiss , Trends Biochem. Sci. 2018, 43, 108. 29269020 10.1016/j.tibs.2017.11.008 PMC5801066

[21] D. R. Myers , J. Zikherman , J. P. Roose , Trends Immunol. 2017, 38, 844. 28754596 10.1016/j.it.2017.06.010 PMC5669999

[22] J. Eggert , W. M. Zinzow‐Kramer , Y. Hu , E. M. Kolawole , Y.‐L. Tsai , A. Weiss , B. D. Evavold , K. Salaita , C. D. Scharer , B. B. Au‐Yeung , Sci. Signal. 2024, 17, adh0439. 10.1126/scisignal.adh0439 PMC10897907 38319998

[23] K. Kelly , U. Siebenlist , Curr. Opin. Immunol. 1995, 7, 327. 7546396 10.1016/0952-7915(95)80106-5

[24] D. R. Myers , T. Lau , E. Markegard , H. W. Lim , H. Kasler , M. Zhu , A. Barczak , J. P. Huizar , J. Zikherman , D. J. Erle , W. Zhang , E. Verdin , J. P. Roose , Cell Rep. 2017, 19, 1558. 28538176 10.1016/j.celrep.2017.04.076 PMC5587137

[25] E. Jennings , T. A. E. Elliot , N. Thawait , S. Kanabar , J. C. Yam‐Puc , M. Ono , K.‐M. Toellner , D. C. Wraith , G. Anderson , D. Bending , Cell Rep. 2020, 33, 108328. 33147449 10.1016/j.celrep.2020.108328 PMC7653457

[26] S. M. Gadgeel , N. A. Pennell , M. J. Fidler , B. Halmos , P. Bonomi , J. Stevenson , B. Schneider , A. Sukari , J. Ventimiglia , W. Chen , C. Galasso , A. Wozniak , J. Boerner , G. P. Kalemkerian , J. Thorac. Oncol. 2018, 13, 1393. 29775808 10.1016/j.jtho.2018.05.002 PMC6833950

[27] H. Yu , C. Batenchuk , A. Badzio , T. A. Boyle , P. Czapiewski , D. C. Chan , X. Lu , D. Gao , K. Ellison , A. A. Kowalewski , C. J. Rivard , R. Dziadziuszko , C. Zhou , M. Hussein , D. Richards , S. Wilks , M. Monte , W. Edenfield , J. Goldschmidt , R. Page , B. Ulrich , D. Waterhouse , S. Close , J. Jassem , K. Kulig , F. R. Hirsch , J. Thorac. Oncol. 2017, 12, 110. 27639678 10.1016/j.jtho.2016.09.002 PMC5353355

[28] H. S. Azzam , J. B. DeJarnette , K. Huang , R. Emmons , C.‐S. Park , C. L. Sommers , D. El‐Khoury , E. W. Shores , P. E. Love , J. Immunol. 2001, 166, 5464. 11313384 10.4049/jimmunol.166.9.5464

[29] G. Voisinne , A. Gonzalez de Peredo , R. Roncagalli , Front. Immunol. 2018, 9, 2900. 30581443 10.3389/fimmu.2018.02900 PMC6292949

[30] J. F. Ashouri , A. Weiss , J. Immunol. 2017, 198, 657. 27940659 10.4049/jimmunol.1601301 PMC5224971

[31] M. Yang , M. Cui , Y. Sun , S. Liu , W. Jiang , Cell Commun. Signal. 2024, 22, 338. 38898505 10.1186/s12964-024-01711-w PMC11186190

[32] W. Chen , Y. Liao , P. Sun , J. Tu , Y. Zou , J. Fang , Z. Chen , H. Li , J. Chen , Y. Peng , L. Wen , X. Xie , J. Transl. Med. 2024, 22, 66. 38229155 10.1186/s12967-023-04794-0 PMC10792867

[33] Z. Cao , D. Xu , J. Harding , W. Chen , X. Liu , Z. Wang , L. Wang , T. Qi , S. Chen , X. Guo , I. S. Y. Chen , J. Guo , Y. Lu , J. Wen , Sci. Transl. Med. 2023, 15, add2712. 10.1126/scitranslmed.add2712 PMC10720694 37820006

[34] M. L. Frank , K. Lu , C. Erdogan , Y. Han , J. Hu , T. Wang , J. V. Heymach , J. Zhang , A. Reuben , Clin. Cancer Res. 2023, 29, 994. 36413126 10.1158/1078-0432.CCR-22-2469 PMC10011887

[35] R. Vazquez‐Lombardi , J. S. Jung , F. S. Schlatter , A. Mei , N. R. Mantuano , F. Bieberich , K.‐L. Hong , J. Kucharczyk , E. Kapetanovic , E. Aznauryan , C. R. Weber , A. Zippelius , H. Läubli , S. T. Reddy , Immunity 2022, 55, 1953. 36174557 10.1016/j.immuni.2022.09.004

[36] A.‐N. Chen , Y. Luo , Y.‐H. Yang , J.‐T. Fu , X.‐M. Geng , J.‐P. Shi , J. Yang , Front. Immunol. 2021, 12, 688910. 34177945 10.3389/fimmu.2021.688910 PMC8222712

[37] L. Chen , L. Huang , Y. Gu , W. Cang , P. Sun , Y. Xiang , Int. J. Mol. Sci. 2022, 23, 11943. 36233246 10.3390/ijms231911943 PMC9569569

[38] T. Sekiya , S. Hidano , S. Takaki , Cell Rep. 2024, 43, 11943. 10.1016/j.celrep.2024.113954 38492221

[39] J. Eggert , B. B. Au‐Yeung , Curr. Opin. Immunol. 2021, 73, 43. 34653787 10.1016/j.coi.2021.09.007 PMC8648992

[40] H. Wang , J. Holst , S.‐R. Woo , C. Guy , M. Bettini , Y. Wang , A. Shafer , M. Naramura , M. Mingueneau , L. L. Dragone , S. M. Hayes , B. Malissen , H. Band , D. A. A. Vignali , EMBO J. 2010, 29, 1285. 20150895 10.1038/emboj.2010.10 PMC2857457

[41] J. Qu , P. Li , Z. Sun , Front. Immunol. 2023, 14, 1284344. 37965331 10.3389/fimmu.2023.1284344 PMC10641494

[42] M. V. Liberti , J. W. Locasale , Trends Biochem. Sci. 2020, 45, 179. 31901298 10.1016/j.tibs.2019.12.004

[43] Y. Zhang , H. Song , M. Li , P. Lu , Clin. Transl. Med. 2024, 14, 1614. 10.1002/ctm2.1614 PMC10921234 38456209

[44] L. Wu , Y. Jin , X. Zhao , K. Tang , Y. Zhao , L. Tong , X. Yu , K. Xiong , C. Luo , J. Zhu , F. Wang , Z. Zeng , D. Pan , Cell Metab. 2023, 35, 1580. 37506695 10.1016/j.cmet.2023.07.001

[45] T. Qiao , Y. Xiong , Y. Feng , W. Guo , Y. Zhou , J. Zhao , T. Jiang , C. Shi , Y. Han , Front. Oncol. 2021, 11, 632364. 33859941 10.3389/fonc.2021.632364 PMC8042335

[46] Y. Fang , W. Liu , Z. Tang , X. Ji , Y. Zhou , S. Song , M. Tian , C. Tao , R. Huang , G. Zhu , X. Jiang , J. Gao , W. Qu , H. Wang , P. Zhou , X. Wu , L. Jin , H. Sun , Z. Ding , Y. Peng , S. Zhao , J. Zhou , J. Fan , W. Xu , Y. Shi , Hepatology 2023, 77, 109. 35043976 10.1002/hep.32348

[47] W. Li , C. Zhou , L. Yu , Z. Hou , H. Liu , L. Kong , Y. Xu , J. He , J. Lan , Q. Ou , Y. Fang , Z. Lu , X. Wu , Z. Pan , J. Peng , J. Lin , Autophagy 2024, 20, 114. 37615625 10.1080/15548627.2023.2249762 PMC10761097

[48] D. Raychaudhuri , P. Singh , B. Chakraborty , M. Hennessey , A. J. Tannir , S. Byregowda , S. M. Natarajan , A. Trujillo‐Ocampo , J. S. Im , S. Goswami , Nat. Immunol. 2024, 25, 2140. 39375549 10.1038/s41590-024-01985-9 PMC13211864

[49] S. V. Liu , M. Reck , A. S. Mansfield , T. Mok , A. Scherpereel , N. Reinmuth , M. C. Garassino , J. De Castro Carpeno , R. Califano , M. Nishio , F. Orlandi , J. Alatorre‐Alexander , T. Leal , Y. Cheng , J.‐S. Lee , S. Lam , M. McCleland , Y. Deng , S. Phan , L. Horn , J. Clin. Oncol. 2021, 39, 619. 33439693 10.1200/JCO.20.01055 PMC8078320

[50] J. S. Lim , A. Ibaseta , M. M. Fischer , B. Cancilla , G. O'Young , S. Cristea , V. C. Luca , D. Yang , N. S. Jahchan , C. Hamard , M. Antoine , M. Wislez , C. Kong , J. Cain , Y.‐W. Liu , A. M. Kapoun , K. C. Garcia , T. Hoey , C. L. Murriel , J. Sage , Nature 2017, 545, 360. 28489825 10.1038/nature22323 PMC5776014

[51] B. Chen , M. S. Khodadoust , C. L. Liu , A. M. Newman , A. A. Alizadeh , Cancer Systems Biology, Methods in Molecular Biology, Vol. 1711, Humana Press, New York, NY 2018.

