# Supplementary material for: The LDH‐H3K18La‐Nur77 Axis Potentiates Immune Escape in Small Cell Lung Cancer
Source: Adv Sci (Weinh). 2025 Jun 24;12(34):e13608. doi: 10.1002/advs.202413608 (PMC12442664; doi:10.1002/advs.202413608)
Supplement: Supplementary file 1 — Supporting Information [file ADVS-12-e13608-s001.docx]

**Title: The LDH-H3K18La-Nur77 Axis Potentiates Immune Escape in Small Cell Lung Cancer**

**Author order:** Xiaoling Shang ^1#^, Bo Cheng ^2#^, Chenyue Zhang ^3^, Chenglong Zhao ^4^, Ruiqing Wang^2^, Xun Zhang^5^, Dizhi Jiang^2^, Xinyu Zhang^2^, Xinyue Ma^2^, Hongyuan Mao^2^, Zerun Li^2^, Chenhan Huang^2^, Tianzi Wang^2^, Kaiyue Guo ^2^, Liwen Wang ^1^, Ning Tang ^1^, Haiyong Wang ^1*^

**Affiliations:**

^1^ Department of internal Medicine-Oncology, Shandong Cancer Hospital and Institute, Shandong First Medical University and Shandong Academy of Medical Sciences, Jinan 250017, China.

^2^ Department of Radiation Oncology, Qilu Hospital of Shandong University, 107 Wenhuaxi Road, Jinan 250012, China.

^3^ Department of Integrated Therapy, Fudan University Shanghai Cancer Center, Shanghai Medical College, Shanghai, China.

^4^ Department of Pathology, The First Affiliated Hospital of Shandong First Medical University and Shandong Provincial Qianfoshan Hospital, Jinan, Shandong, China.

^5^ Department of Medical Oncology, University of Groningen, University Medical Center Groningen, Hanzeplein 1, 9713 GZ Groningen, The Netherlands.

***Corresponding authors:**

Haiyong Wang

Department of internal Medicine-Oncology, Shandong Cancer Hospital and Institute, Shandong First Medical University and Shandong Academy of Medical Sciences, Jinan 250117, China.

Tel: 860531-87984777; Fax: 86531-87984079;

E-mail: [wanghaiyong6688@126.com](mailto:wanghaiyong6688@126.com) for Haiyong Wang

**^#^** These authors have contributed equally to this work.

This file includes:

Fig. S1-S10

Table S1-S12

**
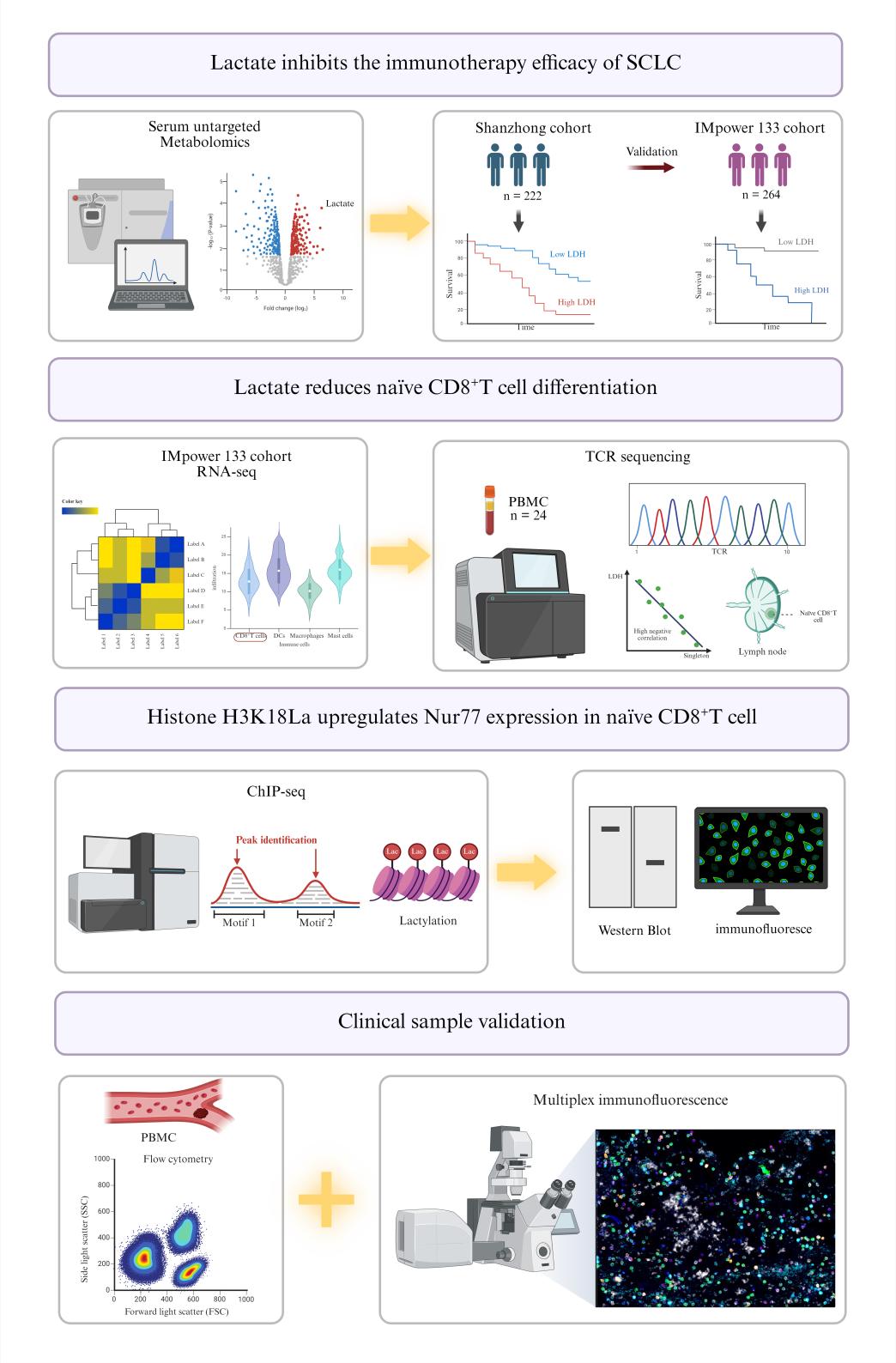
Figure S1**

**Figure S1. The study design of this work.** This schematic summarizes our mechanistic study on lactate-mediated immune escape in small cell lung cancer (SCLC). Metabolomic profiling initially identified aberrant lactate accumulation in SCLC patients, which was subsequently validated in Shanzhong cohort and IMpower133 cohort, establishing lactate dehydrogenase (LDH) as a critical factor of immunotherapy response. Multi-omics analyses revealed that lactate suppresses naïve CD8^+^ T cell differentiation, thereby facilitating tumor immune escape and diminishing anti-PD-1 efficacy. At the molecular level, we discovered that histone H3K18 lactylation epigenetically upregulates Nur77 expression in naïve CD8^+^ T cells, leading to impaired T cell function. These findings were further confirmed through comprehensive clinical validation using patient-derived samples. Our study delineates how lactate orchestrates immune escape in SCLC by modulating metabolic-epigenetic crosstalk in CD8^+^ T cells.

**
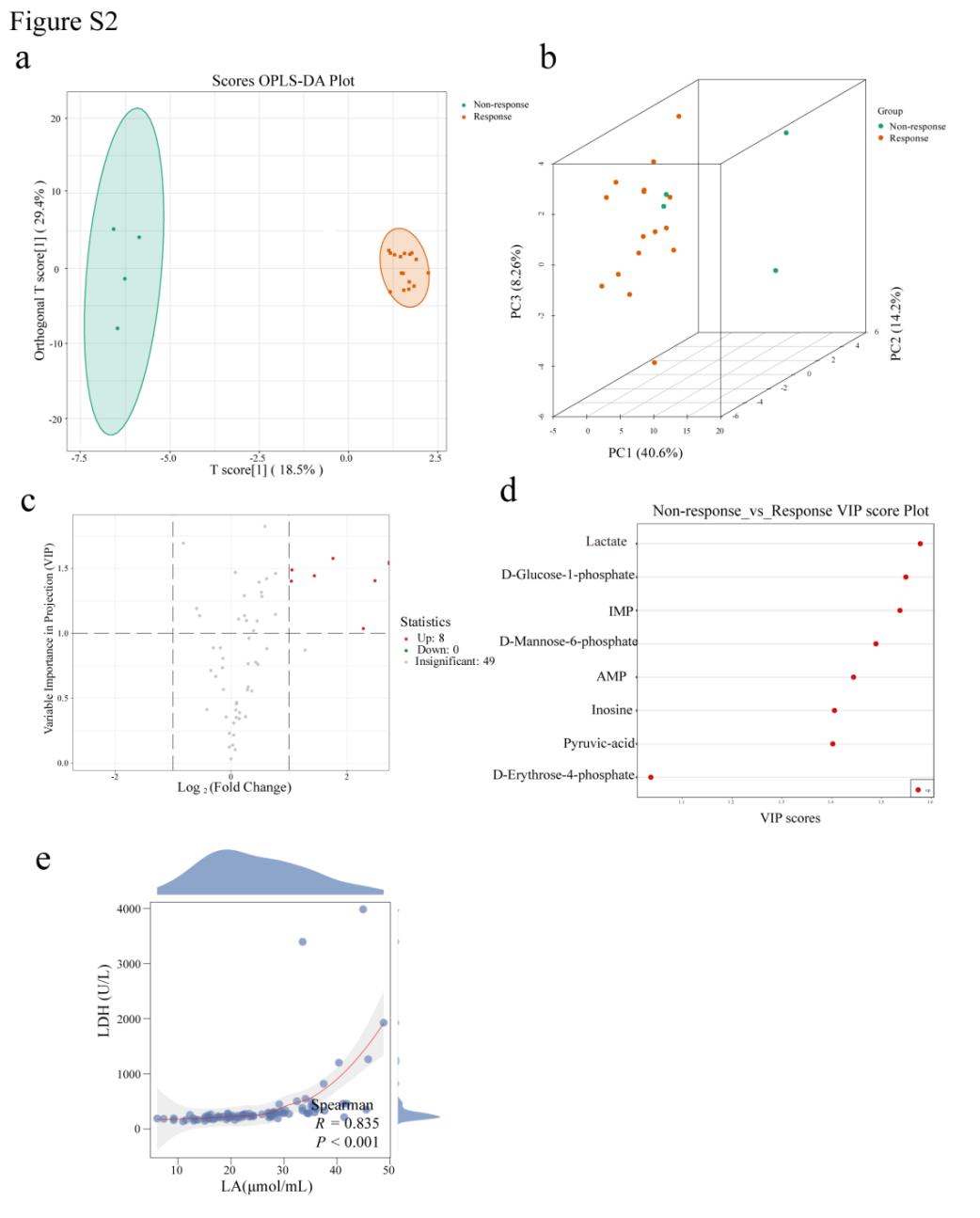
Figure S2**

**Figure S2. The Role of Lactate Metabolism in Modulating Immunotherapy Outcomes. a** The Orthogonal Partial Least Squares Discriminant Analysis (OPLS-DA) scores of plasma-targeted metabolomics data comparing responsers (n=16) and non-responsers (n=4) in SCLC patients. **b** A 3-D Principal Component Analysis (PCA) of plasma-targeted metabolomics data comparing responsers and non-responsers in SCLC patients. **c** Volcano plot of the detected metabolites in plasma metabolomics (non-responser versus responsers). **d** The VIP score plot of identified differential metabolites. **e** Correlation analysis between serum lactate levels and LDH activity in 90 treatment-naive SCLC patients receiving first-line anti-PD-1/PD-L1 combined with EP regimen (Spearman R = 0.835, *P* < 0.001), with regression line shown (shaded area represents 95% CI).

**Figure S3**

**
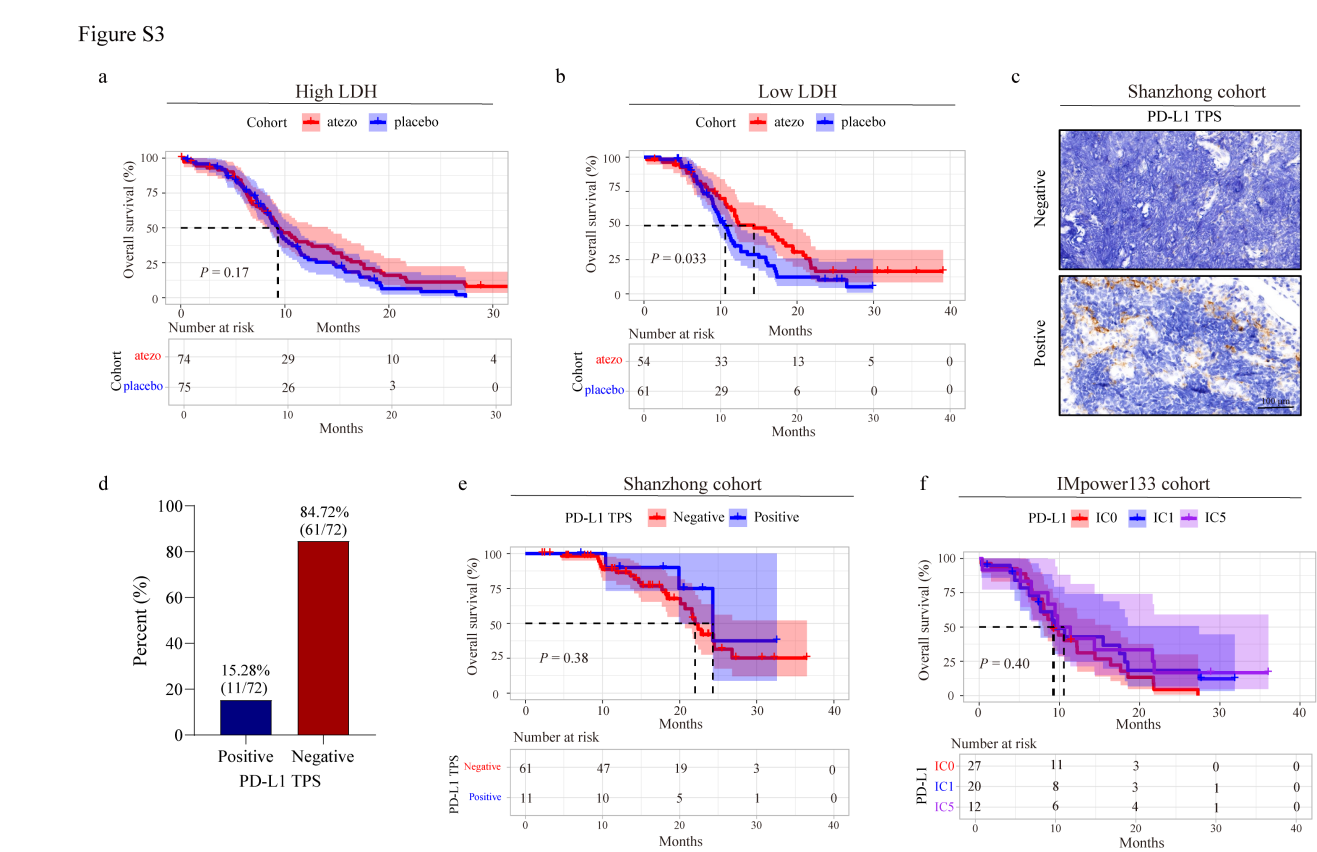
**

**Figure S3. Survival outcomes and PD-L1 expression analysis in extensive-stage small cell lung cancer (ES-SCLC) patients treated with immunotherapy. a** Kaplan-Meier curve comparing overall survival (OS) in ES-SCLC patients with high LDH levels from the IMpower133 cohort receiving atezolizumab versus chemotherapy (*P* = 0.17). **b** Kaplan-Meier curve comparing OS in ES-SCLC patients with low LDH levels from the IMpower133 cohort treated with atezolizumab versus chemotherapy (*P* = 0.033). **c** Representative immunohistochemical images of PD-L1 tumor proportion score (TPS)-negative and -positive SCLC tissues from Shanzhong cohort (scale bar: 100 μm). **d** Quantitative analysis of PD-L1 TPS in 72 SCLC patients. **e** Kaplan-Meier curve comparing OS between PD-L1 TPS-negative and -positive SCLC patients from the Shandong cohort (n=72) treated with immunotherapy. **e** Kaplan-Meier curve assessing the association between PD-L1 expression levels and OS in 59 atezolizumab-treated patients from the IMpower133 cohort.

**
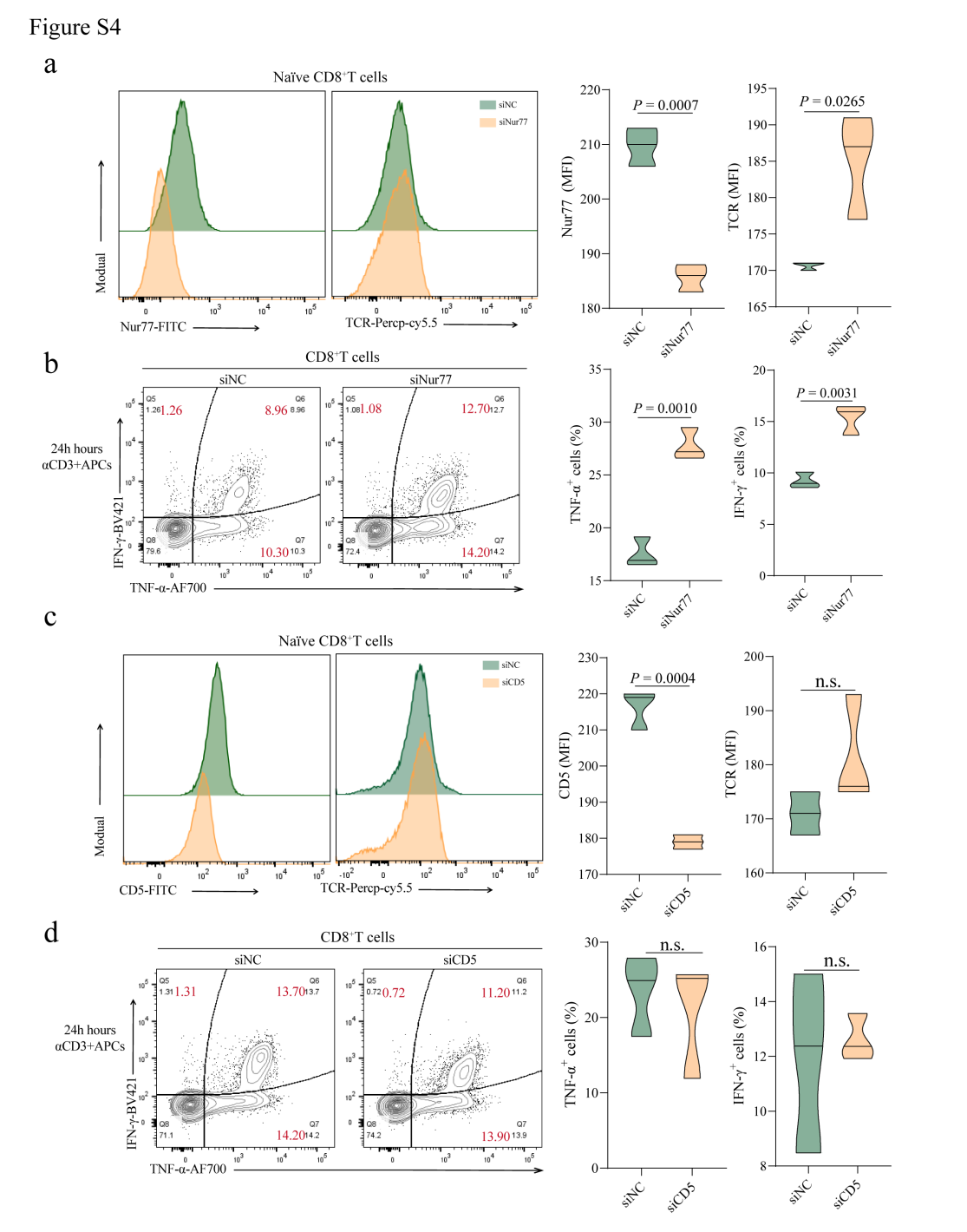
Figure S4**

**Figure S4. Effects of Nur77 and CD5 knockdown on TCR signaling and cytokine production in naïve CD8^+^ T cells. a** Impact of Nur77 knockdown in naïve CD8^+^ T cells on Nur77 and TCR expression levels. **b** Flow cytometry analysis of TNF-α and IFN-γ production in Nur77-knockdown naïve CD8^+^ T cells stimulated for 24 hours with anti-CD3 and APCs. **c** Effect of CD5 knockdown in naïve CD8^+^ T cells on CD5 and TCR expression. **d** Flow cytometry analysis of TNF-α and IFN-γ production in CD5-knockdown naïve CD8^+^ T cells stimulated for 24 hours with anti-CD3 and APCs.

**
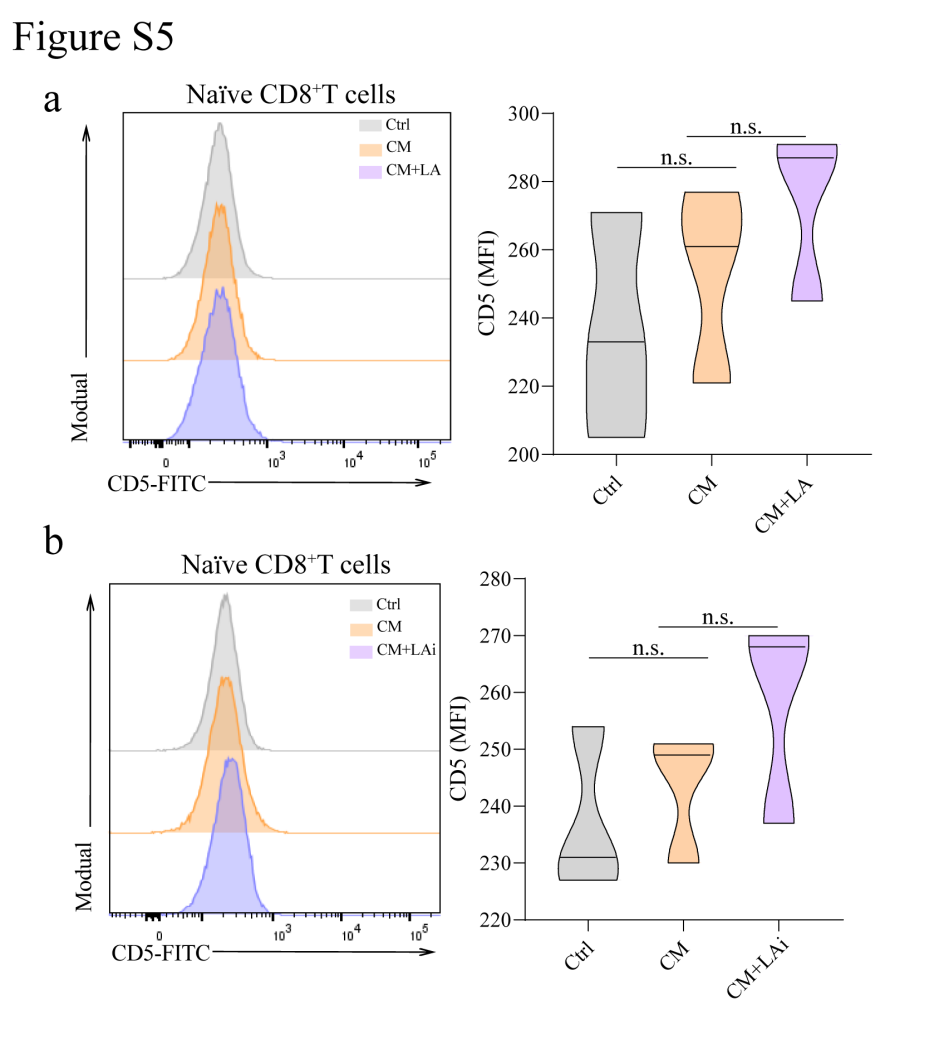
Figure S5**

**Figure S5. Effects of CD5 expression on naïve CD8^+^ T cells in the presence of lactate or lactate inhibitor (LAi). a** Flow cytometry analysis of CD5 expression in naïve CD8^+^ T cells after exposure to CM from H446 with or without lactate. **b** Flow cytometry analysis of CD5 expression in naïve CD8^+^ T cells after exposure to CM from H446 with or without LAi.

**Figure S6**


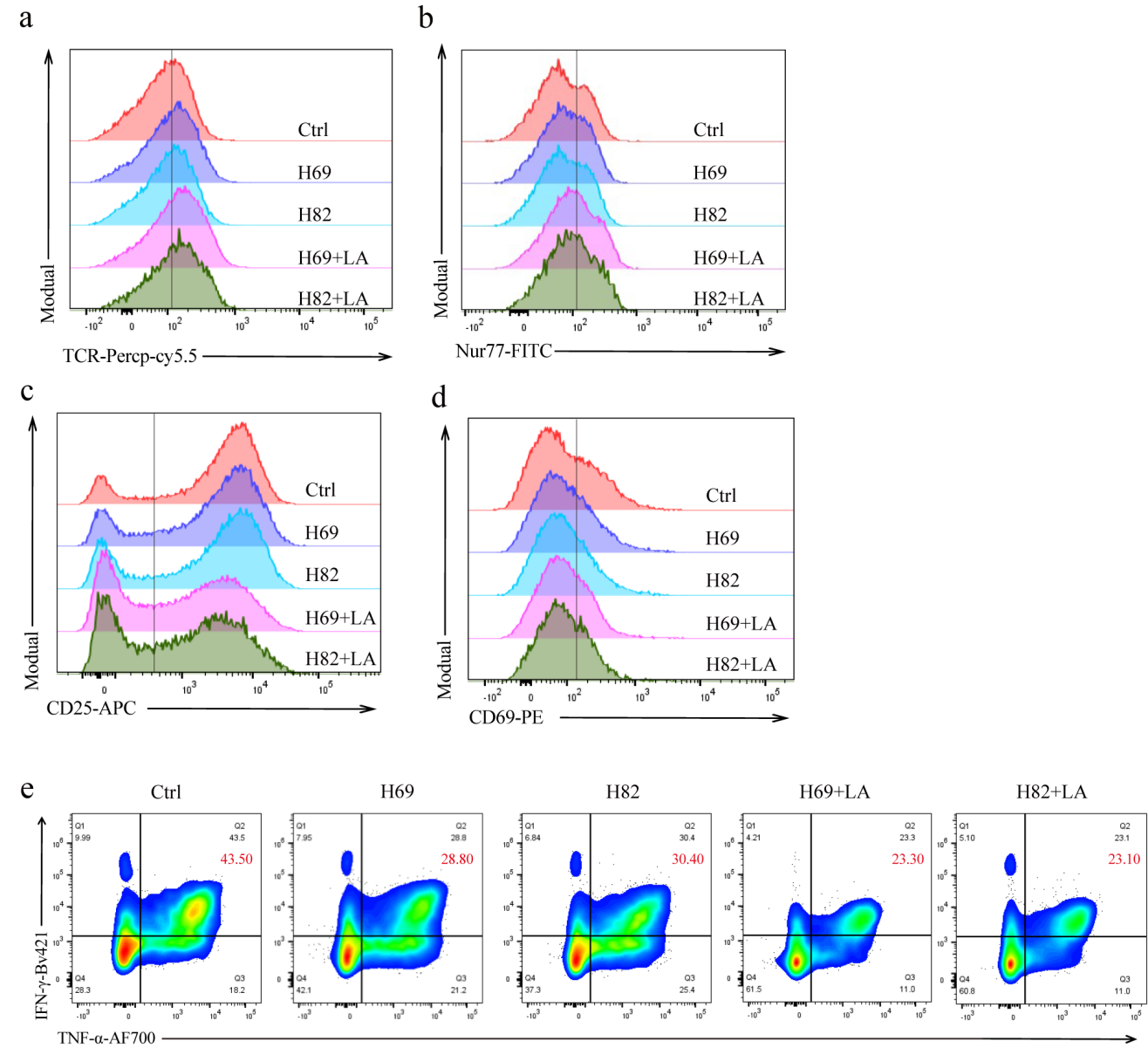


**Figure S6. Effects of tonic TCR signaling on naïve CD8^+^ T cells in the presence of lactate. a-b** Flow cytometry analysis of Nur77 and TCR expression in naïve CD8^+^ T cells after exposure to CM from H69, H82 with or without lactate. **c-d** Histograms show expression of the indicated activation markers (CD25, CD69) of cells stimulated for 24 hours with anti-CD3 and APCs in CD8^+^ T cells after exposure to CM from H69, H82 with or without lactate. **e** Contour plots depict the secretion of IFN-γ and TNF-α by CD8^+^ T cells under H69, H82 CM with or without lactate.

**
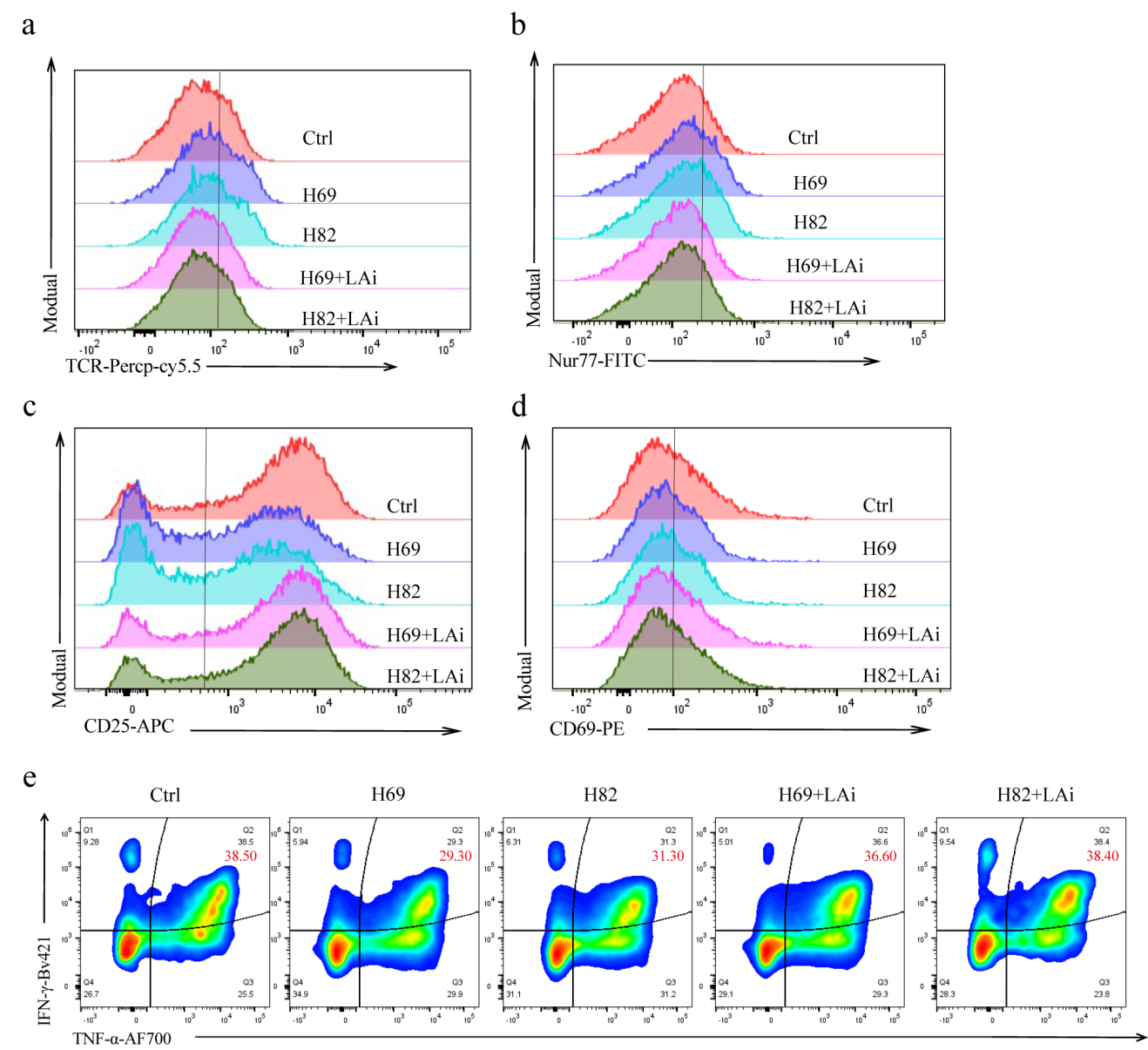
Figure S7**

**Figure S7. Impact of lactate inhibition (LAi) on Nur77 expression and functional characteristics of naïve CD8^+^ T cells. a-b** Flow cytometry analysis of Nur77 and TCR expression in naïve CD8^+^ T cells after exposure to CM from H69, H82 with or without LAi. **c-d** Histograms show expression of the indicated activation markers (CD25, CD69) of cells stimulated for 24 hours with anti-CD3 and APCs in CD8^+^ T cells after exposure to CM from H69, H82 with or without LAi. **e** Contour plots depict the secretion of IFN-γ and TNF-α by CD8^+^ T cells under H69, H82 CM with or without LAi.


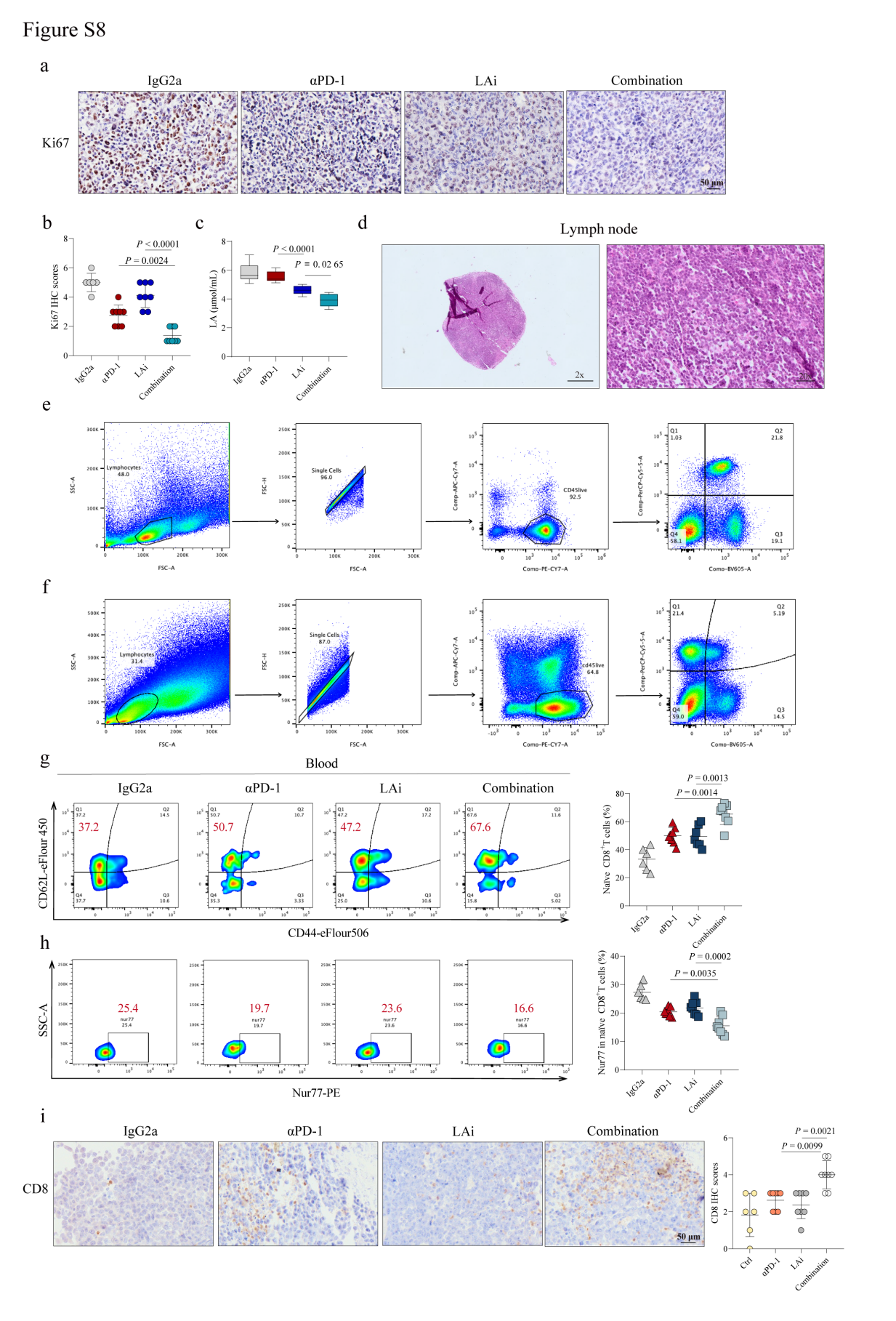
**Figure S8**

**Figure S8. Effects of lactate inhibitor on naïve CD8^+^ T cells and Nur77 expression. a** Representative IHC staining with Ki-67 in KP3 tumors with different treatments were shown. Scale bar, 50 μm. **b** Bar graphs depict Ki67 scores for IHC staining. **c** Lactate content in different groups of KP3 tumors was measured. **d** H&E staining of mediastinal lymph nodes from terminal-stage mice. **e** Gate strategy for lymph node flow cytometry analysis in mice. **f** Gate strategy for tumor tissue flow cytometry analysis in mice. **g** Flow cytometry of naïve CD8^+^ T cells (CD44^low^CD62L^high^) in PB of mice. Cells were gated on viable CD8^+^ T cells. **h** Flow cytometry of Nur77 expression on naïve CD8^+^ T cells in PB of mice. **i** Representative IHC staining with CD8 in KP3 tumors with different treatments were shown. Scale bar, 50 μm. Bar graphs depict CD8 scores for IHC staining.

**Figure S9**


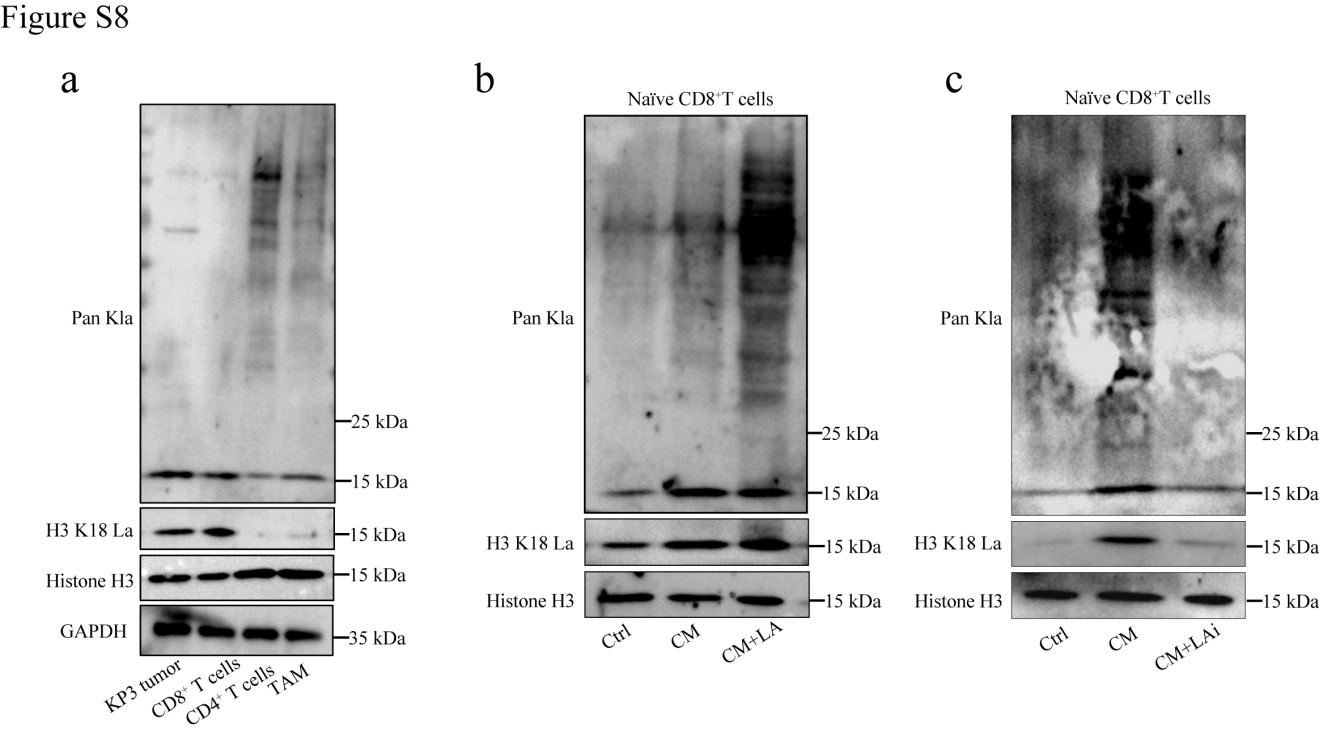


**Figure S9. Histone Lactylation. a** Detection of lactylation modification levels in various cell types within the KP3 tumor microenvironment. **b** Immunblot analysis of PanKla and H3K18La levels in naïve CD8^+^ T cells under H82 CM with or without lactate. **c** Immunblot analysis of PanKla and H3K18La levels in naïve CD8^+^ T cells under H82 CM with or without LAi.

**Figure S10**


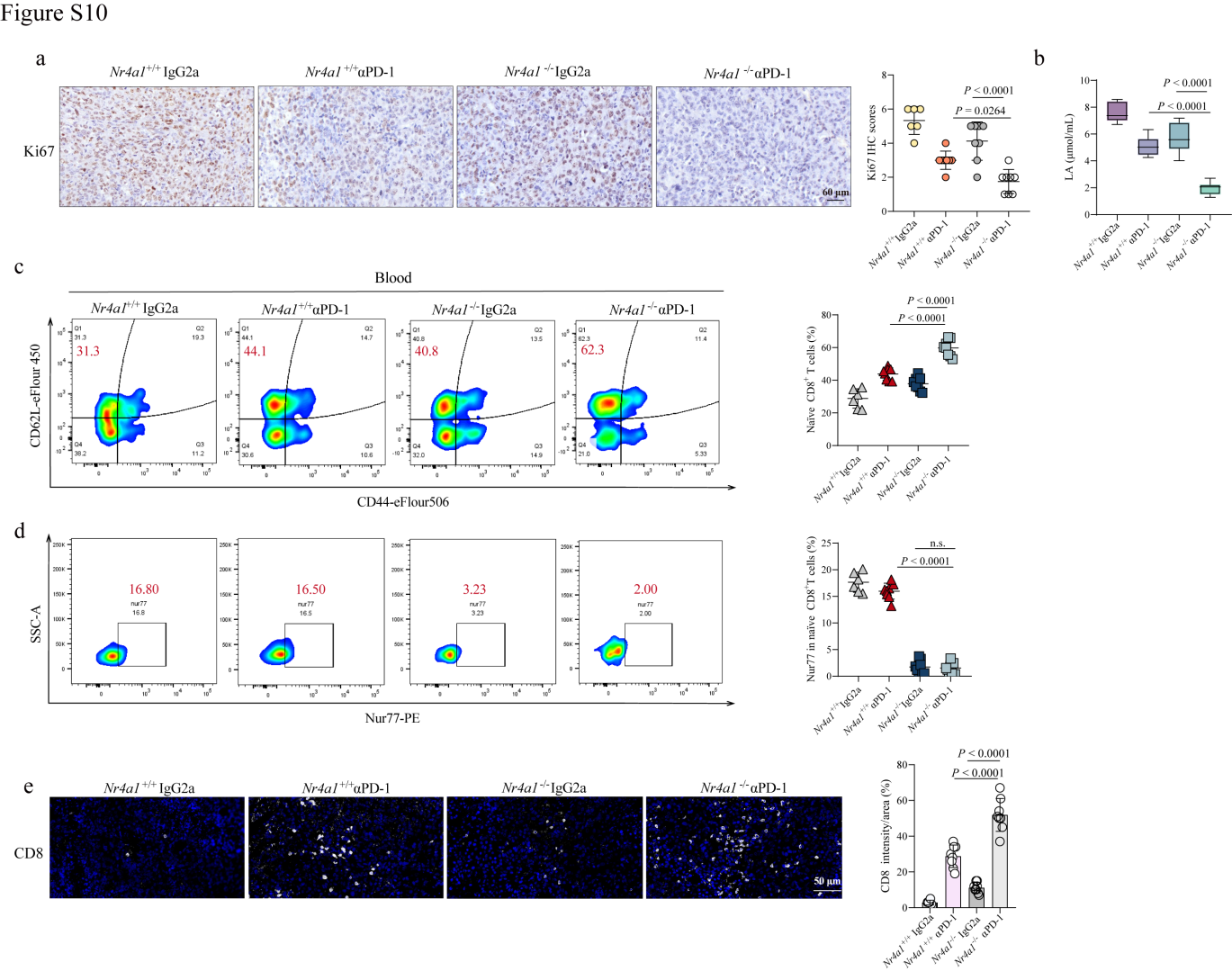


**Figure S10. Impact of Nr4a1 knockout on CD8^+^ T cells and TME. a** Representative IHC staining with Ki-67 in KP3 tumors with different treatments were shown. Scale bar, 50 μm. **b** Lactate content in different groups of KP3 tumors was measured. **c** Flow cytometry of naïve CD8^+^ T cells (CD44^low^CD62L^high^) in PB of mice. Cells were gated on viable CD8^+^ T cells. **d** Flow cytometry of Nur77 expression on naïve CD8^+^ T cells in PB of mice. **e** Representative Immunofluorescence staining with CD8 in KP3 tumors with different treatments were shown. Scale bar, 50 μm.

**Table S1. Lactate and LDH levels in the serum of SCLC patients from Shandong Cancer Hospital and Institute cohort.**

| **ID** | **LDH (U/L)** | | **LA（umol/mL）** | **First-line treatment** |
| --- | --- | --- | --- | --- |
| P1 | 302 | 35.78105651 | | Durvalumab+EP |
| P2 | 252 | 22.13255068 | | Atezolizumab+EP |
| P3 | 1203 | 40.34928411 | | Atezolizumab+EP |
| P4 | 210 | 21.16815068 | | Durvalumab+EP |
| P5 | 232 | 19.88475945 | | Serplulimab+EP |
| P6 | 222 | 22.30928832 | | Serplulimab+EP |
| P7 | 140 | 10.96777594 | | Durvalumab+EP |
| P8 | 227 | 23.45109273 | | Atezolizumab+EP |
| P9 | 822 | 37.47145283 | | Serplulimab+EP |
| P10 | 408 | 35.10476275 | | Durvalumab+EP |
| P11 | 3395 | 33.50883698 | | Serplulimab+EP |
| P12 | 448 | 29.10986078 | | Durvalumab+EP |
| P13 | 256 | 12.27329213 | | Atezolizumab+EP |
| P14 | 266 | 19.43510292 | | Tislelizumab+EP |
| P15 | 224 | 15.89248722 | | Durvalumab+EP |
| P16 | 276 | 22.68939761 | | Adebrelimab+EP |
| P17 | 245 | 27.37618277 | | Atezolizumab+EP |
| P18 | 236 | 27.62055605 | | Tislelizumab+EP |
| P19 | 228 | 18.53952394 | | Atezolizumab+EP |
| P20 | 198 | 15.78028508 | | Durvalumab+EP |
| P21 | 165 | 11.52026924 | | Tislelizumab+EP |
| P22 | 303 | 28.34557798 | | Serplulimab+EP |
| P23 | 216 | 19.31015637 | | Atezolizumab+EP |
| P24 | 385 | 33.45376238 | | Durvalumab+EP |
| P25 | 233 | 24.23934583 | | Atezolizumab+EP |
| P26 | 222 | 27.61866919 | | Tislelizumab+EP |
| P27 | 1928 | 48.78547496 | | Atezolizumab+EP |
| P28 | 274 | 30.96067481 | | Atezolizumab+EP |
| P29 | 351 | 45.51881257 | | Durvalumab+EP |
| P30 | 3984 | 44.91888909 | | Tislelizumab+EP |
| P31 | 201 | 21.55024097 | | Atezolizumab+EP |
| P32 | 299 | 29.69302052 | | Serplulimab+EP |
| P33 | 229 | 23.78349514 | | Serplulimab+EP |
| P34 | 248 | 15.67620894 | | Atezolizumab+EP |
| P35 | 197 | 12.84648931 | | Tislelizumab+EP |
| P36 | 215 | 41.33480698 | | Durvalumab+EP |
| P37 | 495 | 35.24609963 | | Serplulimab+EP |
| P38 | 226 | 15.19230988 | | Atezolizumab+EP |
| P39 | 151 | 12.96998057 | | Durvalumab+EP |
| P40 | 202 | 19.64298642 | | Serplulimab+EP |
| P41 | 334 | 37.66800555 | | Atezolizumab+EP |
| P42 | 287 | 29.98403652 | | Tislelizumab+EP |
| P43 | 166 | 13.39333297 | | Atezolizumab+EP |
| P44 | 214 | 17.53909089 | | Atezolizumab+EP |
| P45 | 221 | 19.81782225 | | Durvalumab+EP |
| P46 | 187 | 15.97953395 | | Serplulimab+EP |
| P47 | 311 | 28.04418762 | | Adebrelimab+EP |
| P48 | 169 | 13.78593445 | | Atezolizumab+EP |
| P49 | 366 | 36.13528394 | | Serplulimab+EP |
| P50 | 209 | 18.03881538 | | Durvalumab+EP |
| P51 | 266 | 16.21815031 | | Tislelizumab+EP |
| P52 | 405 | 35.49039628 | | Serplulimab+EP |
| P53 | 285 | 34.69928207 | | Atezolizumab+EP |
| P54 | 459 | 41.81066447 | | Atezolizumab+EP |
| P55 | 195 | 9.21536455 | | Atezolizumab+EP |
| P56 | 191 | 6.09421849 | | Tislelizumab+EP |
| P57 | 234 | 17.63475631 | | Durvalumab+EP |
| P58 | 504 | 32.43039503 | | Serplulimab+EP |
| P59 | 297 | 27.02537421 | | Atezolizumab+EP |
| P60 | 193 | 28.83427603 | | Durvalumab+EP |
| P61 | 190 | 15.88335309 | | Atezolizumab+EP |
| P62 | 244 | 20.34798583 | | Durvalumab+EP |
| P63 | 215 | 15.69368948 | | Tislelizumab+EP |
| P64 | 242 | 21.36333211 | | Serplulimab+EP |
| P65 | 548 | 34.04625332 | | Atezolizumab+EP |
| P66 | 188 | 16.93410655 | | Adebrelimab+EP |
| P67 | 278 | 23.46723154 | | Atezolizumab+EP |
| P68 | 288 | 27.88568112 | | Tislelizumab+EP |
| P69 | 189 | 14.99485147 | | Durvalumab+EP |
| P70 | 289 | 27.22080184 | | Adebrelimab+EP |
| P71 | 161 | 9.18962455 | | Atezolizumab+EP |
| P72 | 165 | 20.71443086 | | Adebrelimab+EP |
| P73 | 1265 | 45.87196487 | | Atezolizumab+EP |
| P74 | 210 | 27.16329622 | | Serplulimab+EP |
| P75 | 150 | 15.09211893 | | Tislelizumab+EP |
| P76 | 175 | 19.14601915 | | Durvalumab+EP |
| P77 | 462 | 41.16524346 | | Adebrelimab+EP |
| P78 | 334 | 33.50316904 | | Serplulimab+EP |
| P79 | 245 | 24.21985049 | | Durvalumab+EP |
| P80 | 220 | 20.19268271 | | Atezolizumab+EP |
| P81 | 274 | 28.52297732 | | Tislelizumab+EP |
| P82 | 263 | 22.40309058 | | Durvalumab+EP |
| P83 | 287 | 34.33825668 | | Tislelizumab+EP |
| P84 | 284 | 19.39565265 | | Serplulimab+EP |
| P85 | 258 | 22.25495793 | | Durvalumab+EP |
| P86 | 266 | 25.84049133 | | Tislelizumab+EP |
| P87 | 185 | 7.31654381 | | Atezolizumab+EP |
| P88 | 339 | 30.25747389 | | Adebrelimab+EP |
| P89 | 313 | 29.27040089 | | Durvalumab+EP |
| P90 | 177 | 16.60737325 | | Atezolizumab+EP |

**Table S2. Patients’ characteristics form Shandong Cancer Hospital and Institute.**

| **Variables** | **Number (%)** |
| --- | --- |
| **Sex** |  |
| Male | 185 (83.3) |
| Female | 37 (16.7) |
| **Age** |  |
| < 65 | 128 (57.7) |
| ≥ 65 | 94 (42.3) |
| **Smoking history** |  |
| Yes | 136 (61.3) |
| No | 86 (38.7) |
| **Family history** |  |
| Yes | 33 (14.9) |
| No | 179 (80.6) |
| Others | 10 (4.5) |
| **LDH** |  |
| Low | 194 (87.4) |
| High | 28 (12.6) |

**Table S3. Influence of different variables on overall survival (OS) for patients with SCLC treated with ICIs from Shandong Cancer Hospital and Institute cohort.**

| **Characteristics** | **Univariate analysis** | | **Multivariate analysis** | |
| --- | --- | --- | --- | --- |
|  | **HR (95% CI)** | ***P*** | **HR (95% CI)** | ***P*** |
| **Age** |  |  |  |  |
| < 65 | Reference |  |  |  |
| ≥ 65 | 1.049 (0.674 - 1.634) | 0.831 |  |  |
| **Sex** |  |  |  |  |
| Male | Reference |  | Reference |  |
| Female | 0.472 (0.235 - 0.945) | **0.034** | 0.581 (0.267 - 1.263) | 0.171 |
| **TOBHX** |  |  |  |  |
| Never | Reference |  | Reference |  |
| Curr/Prev | 1.595 (1.000 - 2.544) | 0.050 | 1.255 (0.746 - 2.112) | 0.393 |
| **Family history** |  |  |  |  |
| Yes | Reference |  |  |  |
| No | 1.140 (0.678 - 1.917) | 0.621 |  |  |
| Unknown | 0.845 (0.264 - 2.708) | 0.777 |  |  |
| **LDH** |  |  |  |  |
| Low | Reference |  | Reference |  |
| High | 4.285 (2.349 - 7.817) | **< 0.001** | 4.037 (2.212 - 7.368) | **< 0.001** |

**Table S4. Patients’ characteristics from IMpower133 cohort.**

| **Characteristics** | **Atezo (n = 128)** | **Non-Atezo (n = 136)** | ***P*** |
| --- | --- | --- | --- |
| **Age, n (%)** |  |  | 0.764 |
| < 65 | 72 (27.3%) | 74 (28.0%) |  |
| ≥ 65 | 56 (21.2%) | 62 (23.5%) |  |
| **Sex, n (%)** |  |  | 0.309 |
| Male | 84 (31.8%) | 81 (30.7%) |  |
| Female | 44 (16.7%) | 55 (20.8%) |  |
| **Race, n (%)** |  |  | 0.899 |
| Asian | 106 (40.2%) | 110 (41.7%) |  |
| White | 19 (7.2%) | 23 (8.7%) |  |
| Others | 3 (1.1%) | 3 (1.1%) |  |
| **TOBHX, n (%)** |  |  | 0.244 |
| Cur/Prev | 122 (46.2%) | 134 (50.8%) |  |
| Never | 6 (2.3%) | 2 (0.8%) |  |
| **ECOG, n (%)** |  |  | 0.804 |
| 0 | 48 (18.2%) | 49 (18.6%) |  |
| 1 | 80 (30.3%) | 87 (33.0%) |  |
| **LDH, n (%)** |  |  | 0.662 |
| Low | 54 (20.5%) | 61 (23.1%) |  |
| High | 74 (28.0%) | 75 (28.4%) |  |
| **Number of metastasis sites, n (%)** |  |  | 0.392 |
| < 3 | 39 (14.8%) | 35 (13.3%) |  |
| ≥ 3 | 89 (33.7%) | 101 (38.3%) |  |

**Table S5. Influence of different variables on overall survival (OS) for patients with SCLC treated with atezolizumab from IMpower133 cohort.**

| **Characteristics** | **Univariate analysis** | | **Multivariate analysis** | |
| --- | --- | --- | --- | --- |
|  | **HR (95% CI)** | ***P*** | **HR (95% CI)** | ***P*** |
| **Age** |  |  |  |  |
| < 65 | Reference |  |  |  |
| ≥ 65 | 1.058 (0.713 - 1.571) | 0.779 |  |  |
| **Sex** |  |  |  |  |
| Male | Reference |  |  |  |
| Female | 0.910 (0.598 - 1.387) | 0.661 |  |  |
| **Race** |  |  |  |  |
| White | Reference |  |  |  |
| Asian | 0.879 (0.514 - 1.506) | 0.639 |  |  |
| Others | 1.371 (0.332 - 5.651) | 0.663 |  |  |
| **TOBHX** |  |  |  |  |
| Curr/Prev | Reference |  |  |  |
| Never | 1.221 (0.386 - 3.867) | 0.734 |  |  |
| **ECOG** |  |  |  |  |
| 0 | Reference |  |  |  |
| 1 | 1.072 (0.713 - 1.613) | 0.739 |  |  |
| **Number of metastasis sites** |  |  |  |  |
| < 3 | Reference |  | Reference |  |
| ≥ 3 | 1.937 (1.202 - 3.122) | **0.007** | 1.909 (1.181 - 3.084) | **0.008** |
| **LDH** |  |  |  |  |
| Low | Reference |  | Reference |  |
| High | 1.568 (1.047 - 2.347) | **0.029** | 1.534 (1.025 - 2.298) | **0.038** |

**Table S6. Influence of different variables on overall survival (OS) for patients with SCLC in low LDH group from IMpower133 cohort.**

| **Characteristics** | **Univariate analysis** | | **Multivariate analysis** | |
| --- | --- | --- | --- | --- |
|  | **Hazard ratio (95% CI)** | ***P*** | **Hazard ratio (95% CI)** | ***P*** |
| **Cohort** |  |  |  |  |
| Non-Atezo | Reference |  | Reference |  |
| Atezo | 0.630 (0.410 - 0.969) | **0.035** | 0.597 (0.388 - 0.918) | **0.019** |
| **Age** |  |  |  |  |
| <65 | Reference |  |  |  |
| ≥65 | 1.056 (0.692 - 1.612) | 0.801 |  |  |
| **Sex** |  |  |  |  |
| Male | Reference |  |  |  |
| Female | 0.887 (0.570 - 1.382) | 0.597 |  |  |
| **Race** |  |  |  |  |
| White | Reference |  |  |  |
| Asian | 0.829 (0.438 - 1.566) | 0.563 |  |  |
| Others | 3.177 (1.128 - 8.945) | **0.029** |  |  |
| **TOBHX** |  |  |  |  |
| Curr/Prev | Reference |  |  |  |
| Never | 0.548 (0.076 - 3.954) | 0.551 |  |  |
| **ECOG** |  |  |  |  |
| 0 | Reference |  |  |  |
| 1 | 0.886 (0.579 - 1.356) | 0.578 |  |  |
| **Number of metastasis sites** |  |  |  |  |
| < 3 | Reference |  | Reference |  |
| ≥ 3 | 1.780 (1.116 - 2.840) | **0.016** | 1.869 (1.170 - 2.985) | **0.009** |

**Table S7. List of antibodies used for immunoblotting.**

| **Antibody** | **Company** | **Cat No.** | **Dilution ratio** |
| --- | --- | --- | --- |
| GAPDH | Proteintech | 10494-1-AR | 1:5000 |
| Nur77 | Abcam | Ab283264 | 1:1000 |
| Histone H3 | PTMab | PTM-1001RM | 1:1000 |
| L-Lactyl Lysine | PTMab | PTM-1401 | 1:1000 |
| L-Lactyl-Histone H3 (Lys18) | PTMab | PTM-1406RM | 1:1000 |

**Table S8. List of primers used for real-time quantitative PCR.**

| **Gene** | **Sense** | **Anti-sense** |
| --- | --- | --- |
| Nur77 | CCCAGCCAGACTTACGAAGG | GAGGGGAACAACTTCAGGGG |
| GAPDH | AGGGGAGATTCAGTGTGGTG | GGCCTCCAAGGAGTAAGACC |

**Table S9. List of antibodies used for multiplex immunofluorescent (mIF) staining.**

| **Antibody** | **Company** | **Cat No.** | **Dilution ratio** |
| --- | --- | --- | --- |
| CD8 | Abcam | ab237709 | 1:500 |
| CD45RA | Abcam | ab755 | 1:200 |
| CCR7 | Abcam | ab253187 | 1:200 |
| TCR | Abcam | ab318205 | 1:200 |
| Nur77 | Proteintech | 12235-1-AP | 1:500 |
| CD25 | Abcam | ab128955 | 1:200 |

**Table S10. List of antibodies used for immunohistochemical (IHC) staining.**

| **Antibody** | **Company** | **Cat No.** | **Dilution ratio** |
| --- | --- | --- | --- |
| CD8 | Abcam | ab237709 | 1:500 |
| Ki67 | Bioss | bs-23103R | 1:200 |
| L-Lactyl-Histone H3 (Lys18) | PTMab | PTM-1406RM | 1:200 |
| Nur77 | Proteintech | 12235-1-AP | 1:500 |

**Table S11. List of antibodies and reactive dye used for flow cytometry.**

| **Antibody** | **Reactivity** | **Color** | **Company** | **Cat No.** | **Clone** |
| --- | --- | --- | --- | --- | --- |
| CD3 | Human | PE | Biolegend | 300439 | UCTH1 |
| CD3 | Human | APC | Biolegend | 317318 | OKT3 |
| CD8 | Human | FITC | Biolegend | 344704 | SK1 |
| CD8 | Human | [Brilliant Violet 510™](https://www.bioec.cn/product/5f601cf2afef2b4993dd37f5) | Biolegend | 344732 | SK1 |
| CD45RA | Human | PE/Cyanine7 | Biolegend | 304125 | HI100 |
| CCR7 | Human | APC | Biolegend | 353213 | G043H7 |
| TCR α/β | Human | Percp-cy5.5 | Biolegend | 306723 | IP26 |
| Nur77 | Human |  | Abcam | Ab283264 |  |
| TNF-α | Human | [Alexa Fluor® 700](https://www.bioec.cn/product/5f601ce2afef2b4993dccd23) | Biolegend | 502927 | MAb11 |
| IFN-γ | Human | [Brilliant Violet 421™](https://www.bioec.cn/product/5f601d00afef2b4993ddc968) | Biolegend | 506537 | B27 |
| CD69 | Human | PE | Biolegend | 310906 | FN50 |
| CD25 | Human | APC | Biolegend | 356110 | M-251 |
| CD45 | Mouse | PE/Cyanine7 | Biolegend | 147704 | I3/2.3 |
| CD3 | Mouse | [Brilliant Violet 605™](https://www.bioec.cn/product/5f601cecafef2b4993dd064d) | Biolegend | 100237 | 17A2 |
| CD8a | Mouse | PerCP-Cyanine5.5 | eBioscience^TM^ | 45-0081-82 | 53-6.7 |
| CD44 | Mouse | eFluor™ 506 | eBioscience^TM^ | 69-0441-82 | IM7 |
| CD62L | Mouse | eFluor™ 450 | eBioscience^TM^ | 48-0621-82 | MEL-14 |
| [TCR β chain](https://www.bioec.cn/product/5f601cd9afef2b4993dc88d3) | Mouse | [Alexa Fluor® 700](https://www.bioec.cn/product/5f601cd9afef2b4993dc88d3) | Biolegend | 109223 | H57-597 |
| Nur77 | Mouse | PE | eBioscience^TM^ | 12-5965-80 | 12.14 |
| TNF-α | Mouse | FITC | eBioscience^TM^ | 11-7321-82 | MP6-XT22 |
| IFN-γ | Mouse | APC | Biolegend | 505810 | XMG1.2 |
| Viability Dye 780 |  | APC/CY7 | Biogems | 6291000 |  |

**Table S12. The ChIP–qPCR primers for the Nur77 promoter.**

| **Primer** | **Sense** | **Antisense** |
| --- | --- | --- |
| Primer^1#^ | GTAAAACAGCCAGAGTGCAGC | GAGGAAGGGCTGAAGAGGTG |
| Primer^2#^ | GAAGTGCTCAGACACCCTCC | AGGTCTGTTGGTTGGAGTCA |
| Primer^3#^ | AGCTCATGGACTGGGGTTGA | AGGAGGTGTCCCTGAAAGCA |
| Primer^4#^ | ATCTGGTCCACTCAGCAACC | TGCCTTATTTGCCCAGAGACC |
| Primer^5#^ | AGTCAGTAGGGTGGGGGAC | GCTGGGATTACAGGCGTGAG |
| Primer^6#^ | ATATAAAAATTAGCTGGGCGTGGT | GTCTCGCTCTGTTGCCCA |
| Primer^7#^ | TCACCGCAGCCTCAATCTCC | CAGCACTTTGGAAGGCCGAG |
| Primer^8#^ | CCCACACAGATCCTTGGGAC | GCAGAAAGTGGTTCCGGGC |
